# Exploring the neural basis and modulating factors of implicit altercentric spatial perspective-taking with fNIRS

**DOI:** 10.1038/s41598-023-46205-w

**Published:** 2023-11-23

**Authors:** Natania Ang, Birgit Brucker, David Rosenbaum, Martin Lachmair, Thomas Dresler, Ann-Christine Ehlis, Peter Gerjets

**Affiliations:** 1 LEAD Graduate School & Research Network, University of Tübingen, Walter-Simon-Straße 12, 72072 Tübingen, Germany; 2 Department of Psychiatry and Psychotherapy, Tübingen Center for Mental Health, University Hospital Tübingen, Calwerstraße 14, 72076 Tübingen, Germany; 3 Leibniz-Institut für Wissensmedien, Schleichstraße 6, 72076 Tübingen, Germany; 4 Duale Hochschule Baden-Württemberg Villingen-Schwenningen, Karlstraße 29, 78054 Villingen-Schwenningen, Germany; 5 German Center for Mental Health (DZPG), partner site Tübingen, Tübingen, Germany

**Keywords:** Cognitive neuroscience, Social neuroscience, Human behaviour, Neuroscience, Sensorimotor processing, Near-infrared spectroscopy

## Abstract

Humans spontaneously take the perspective of others when encoding spatial information in a scene, especially with agentive action cues present. This functional near-infrared spectroscopy (fNIRS) study explored how action observation influences implicit spatial perspective-taking (SPT) by adapting a left–right spatial judgment task to investigate whether transformation strategies underlying altercentric SPT can be predicted on the basis of cortical activation. Strategies associated with two opposing neurocognitive accounts (embodied versus disembodied) and their proposed neural correlates (human mirror neuron system; hMNS versus cognitive control network; CCN) are hypothesized. Exploratory analyses with 117 subjects uncover an interplay between perspective-taking and post-hoc factor, consistency of selection, in regions alluding to involvement of the CCN. Descriptively, inconsistent altercentric SPT elicited greater activation than consistent altercentric SPT and/or inconsistent egocentric SPT in the left inferior frontal gyrus (IFG), left dorsolateral prefrontal cortex (DLPFC) and left motor cortex (MC), but not the inferior parietal lobules (IPL). Despite the presence of grasping cues, spontaneous embodied strategies were not evident during implicit altercentric SPT. Instead, neural trends in the inconsistent subgroups (22 subjects; 13 altercentric; 9 egocentric) suggest that inconsistency in selection modulates the decision-making process and plausibly taps on deliberate and effortful disembodied strategies driven by the CCN. Implications for future research are discussed.

## Introduction

Spatial perspective-taking (SPT) is an essential cognitive skill to successfully navigate around and interact with the world^1–4^. It demands an ability to identify the positions of persons and objects in the environment and to encode spatial relationships between them^5,6^. Spatial perspectives are commonly calculated in terms of frames of reference—a coordinate system that specifies the location of objects in the environment—and usually conveyed by the prepositions ‘in front’/ ‘behind’/ ‘left’/ ‘right’^7,8^. However, what might, for example, be described as “to the left of the tree” relative to a viewer’s body coordinates on one side of the tree might also be described as “to the right of the tree” by another viewer on the opposite side of the tree. Consequently, making spatial judgments on this left–right-dimension is essentially tied to which encoding perspective is selected: self or other. Thus, achieving effective social interaction not only relies on the key ability to understand that others can have a differing perspective of the same object or scene from our own but also to spontaneously transform our spatial viewpoint to accommodate the competing perspective of another person^9^. In this exploratory functional near-infrared spectroscopy (fNIRS) study, we examine the neural mechanisms underlying an implicit spatial encoding of the other-perspective and assess, in the context of two opposing neurocognitive accounts and their associated neural correlates, whether spatial transformation strategies undertaken (spontaneous versus deliberate) can be predicted and distinguished on the basis of cortical activation.

Although it is not necessary to understand *how* others perceive their surroundings in order to judge *where* items are located in space relative to them^6^, previous research has shown that people keenly adopt the visuospatial perspectives of others when encoding spatial information in a scene^1,10–12^. When tasked to make spatial judgments about the relative location of objects in the absence of other persons in the scene, people tend to assume a spatial perspective from the *egocentric* frame of reference; that is, representing spatial information relative to the position of their own bodies (e.g., ‘on *my* left’)^1,13^. This computation is said to be anchored in biases to reason the world first through one’s own senses^14–18^. However, when someone else is introduced into the scene, adults show a remarkable sensitivity to encode spatial relationships from the other person’s (*altercentric*) frame of reference rather than their own (e.g., ‘on *his/her* left’), even if the altercentric perspective is irrelevant^1,11,19–23^. For example, Tversky and Hard^1^ showed that an actor’s presence and perceived agency in a photographed scene influenced participants’ written responses to a query strictly about spatial relations between depicted objects in the scene. In spite of the actor displaying a different spatial orientation (in a 180° angle opposite from the viewer’s position), a greater proportion of participants described the relative location of the target object from the front-facing actor’s reference frame in conditions featuring the actor than in the no-actor condition. This finding was comparable regardless of whether the actor was seen performing a reaching action or simply looking at the array of objects. Moreover, highlighting the saliency of the actor’s action by phrasing the query in either active or passive action terms (“In relation to the bottle, where does he place the book/where is the book placed?”), compared to static terms (“In relation to the bottle, where is his book/where is the book?”), further resulted in a majority of participants taking the altercentric perspective. This effect was independent of whether reference to the agent was made in each question type or not. At the behavioral level, these results underscore the role that action perception plays in bringing about spontaneous assimilation and selection of the altercentric spatial perspective, even in a non-communicative setting.

At the neural level, past research investigating the underlying brain systems linking action observation and perspective-taking has suggested multiple neurocognitive routes for taking contrastive perspectives^24–28^. For instance, using functional magnetic resonance imaging (fMRI), Mazzarella and colleagues^24^ found overlapping yet distinct neural mechanisms and brain regions for taking explicitly-instructed egocentric versus altercentric perspectives during left–right spatial judgments. By manipulating agent orientation and observed action (reach versus no-reach), taking the altercentric perspective was proposed to rely concurrently on imagined self-rotation and a suppressed tendency to actually move one’s body, evidenced by greater activation in the dorsomedial prefrontal cortex (dmPFC) and left inferior frontal gyrus (IFG) adjacent to the ventral premotor cortex (PMv) with increasing angular disparity between the agent and the participant. On the other hand, when told to stick with the egocentric perspective, inhibition of the agent’s irrelevant visual perspective via the lateral prefrontal cortex was required, as well as stronger activation in the right IFG with increasing agent orientation. Although their results lend support to previous findings that contrastive perspectives engage dissociable neurocognitive routes for different inhibitory processes, their data could not provide definitive brain-based evidence for how action observation leads to spontaneous adoption of the altercentric perspective^1,11^. It still remains to be elucidated how agentive action cues contribute to spontaneous altercentric perspective-taking in the brain and whether brain activity may distinguish alternative spatial transformation strategies for implicitly taking an altercentric perspective.

We set out to investigate these within the context of the following two neurocognitive accounts—embodied versus disembodied. Each account represents different potential perspective transformation strategies and their associated neural bases. Chiefly, we aimed to determine if making left–right spatial judgments from an altercentric reference frame, without explicit instructions to do so, is largely driven by a spontaneous and automatic transformation strategy or a more deliberate and effortful one, as supported by two corresponding networks. Subsequently, upon establishing the supporting network, we aimed to explore how either spontaneous or effortful altercentric processing would compare to egocentric processing in terms of reaction times. This behavioral data might help bolster the neurophysiological findings and potentially shed light on strategies pertaining to either account.

In the embodied account, taking the altercentric perspective engages an imagined rotation of the self, transforming one’s own egocentric reference frame to the other person’s egocentric reference frame^3,4,17,29^. Calculating spatial relations between objects from the altercentric perspective would involve mentally rotating one’s self into the other person’s position and remapping the locations of the objects to the altercentric reference frame (*remapping* hypothesis^30^). Such an egocentric-based transformation into a new spatial location relies on a mental simulation of the sensorimotor mechanisms involved in actual, physical self-rotation^31^, engaging the premotor, parietal, and occipitotemporal cortices^27,28,32,33^. While this process is typically deemed as effortful and prone to increased reaction times (RTs) compared to maintaining an egocentric perspective^3,34^, taking an altercentric perspective could be seen as spontaneous and automatic if the human mirror neuron system (hMNS) is engaged during action observation^9,35,36^.

The hMNS is a neural network underlying both the perception and execution of sensorimotor representations^37–40^. It has been suggested to be largely involved in automatically facilitating the understanding and imitation of goal-directed hand actions performed by others through motor mirroring^39,41,42^. The most commonly hypothesized areas of the hMNS are the PMv, the IFG and the inferior parietal lobule (IPL)^38,42,43^. The left IFG, in particular, could be considered a key region for social perception and understanding of action goals, being the human homolog of the first site of discovery of mirror neurons in macaque monkeys^44^. Inhibition of the left IFG via repetitive transcranial stimulation (rTMS) has also proven disruption in processes attributed to the hMNS^45^. In our previous work^46^, we also found significant increased activation in the left IFG during the observation of corresponding (as opposed to non-corresponding) hand gestures, crucial to promoting better learning outcomes for learners with low visuospatial abilities. Hence, we propose that a SPT scenario depicting human agentive action cues, such as grasping, may trigger an automatic process of embodied simulation by the hMNS, allowing participants to effortlessly mentally rotate into the agent’s position, and thereby make spontaneous left–right spatial judgments from the altercentric perspective. It should be noted, however, that research is still unclear on the extent to which the hMNS might mitigate cognitive costs during the mental rotation process. Therefore, whether an altercentric response via this neurocognitive route should be comparable to an egocentric response remains a hypothesis to be tested, and equal reaction times between the two responses should not be assumed outrightly.

On the other hand, selecting the altercentric perspective under the disembodied account invokes higher-level cognitive control processes unrelated to bodily motoric simulation^2,30,47,48^. Determining spatial locations of objects from the altercentric perspective would engage deliberate and effortful transformation strategies such as active inhibition of interference from one’s own egocentric perspective^2,9^ or an updating of spatial working memory to adjust spatial relations, initially computed from the egocentric perspective, to that from the other perspective instead (*recomputing* hypothesis)^30^. For example, one could transpose ‘left’ and ‘right’ to make up for asymmetries between the two conflicting perspectives^17,47^. Such object-based transformation and inhibitory processes rely on extra cognitive resources within the cognitive control network (CCN), of which the dorsolateral prefrontal cortex (DLPFC) has been well documented to be a critical component^18,24,25,49,50^. Other coactive regions within the CCN consist of the anterior cingulate cortex/pre-supplementary motor area (ACC/pSMA), inferior frontal junction (IFJ), anterior insular cortex (AIC), dorsal premotor cortex (dPMC) and posterior parietal cortex (PPC)^51^. Functional and structural connectivity analyses giving rise to brain atlases have contributed to specifying brain-behavior circuitry and mapping them to function^52–55^. Cognitive control is the primary function of a larger prefrontal cortex (PFC) that can be further divided or organized into six key sub-networks which, whilst specialized functional units, interact amongst each other. The areas associated with the CCN can be found in the fronto-parietal network (DLPFC) and salience/ventral attention network (ACC, IFJ, AIC)^56^. In fact, distinct pathways in the (dorsal) fronto-parietal network can be further defined depending on attentional priorities in egocentric versus object-/other-centered reference frames^57^. For ease of reference, we will henceforth use the term ‘CCN’ to refer to regions of interest in the disembodied account as opposed to those associated with the embodied account.

The DLPFC has domain-general executive functions including attention, monitoring, updating, and top-down regulation of downstream information processing^58^. During response inhibition, in particular, the DLPFC initiates a two-part inhibitory process by evaluating the environment for the need to stop a response, and then sends a signal to the (right-lateralized) IFG to actively put a ‘brake’ on the response where necessary^59–61^. In turn, the IFG further modulates activity in the pSMA and motor cortex^61^. Depending on the strategy undertaken, activity in these components of the CCN would vary. Deliberate manipulation of spatial relations between objects via the disembodied route has been found to be independent and dissociable from the embodied route, and may take place regardless of whether a scenario enacts social interaction or whether a position presenting an alternative perspective is occupied by a person or inanimate object^2^. Hence, in a similar SPT scenario under implicit conditions, it is feasible that participants might use the other person (and/or the outstretched hand) simply as a marker to locate the target object without the need to rotate into their position. Consciously making left–right spatial judgments from the altercentric perspective using such non-embodied strategies, albeit effortful, would activate components of the CCN.

The present fNIRS study conceptually replicated Tversky and Hard’s^1^ behavioral paradigm to explore the implications of action observation on implicit SPT at the neurophysiological level. We attempted to investigate whether spatial transformation strategies underlying implicit altercentric perspective-taking—represented by the two neurocognitive accounts—could be predicted on the basis of cortical activation. Without explicit instruction to consider the altercentric perspective, the decision to spatially judge the relative locations of objects from either an egocentric or altercentric perspective is left entirely to the participant. Unlike the original task which prompted open-ended written spatial descriptions using a single picture stimulus, ours was adapted into a multiple-trial, forced-choice paradigm that was digitally presented and compatible with fNIRS. This allowed us to allocate ‘left’ or ‘right’ responses to corresponding egocentric or altercentric perspectives and avoid ambiguous neutral responses. We adopted the “action-question-no-actor-mentioned” condition which highlights the *action* performed by an agent in the query but omits explicit reference to the agent, aiming to replicate the approximately 50–50% egocentric to altercentric response distribution (excluding the 20% neutral responses) they found^1^. We expected a clearer comparison of cortical activation between the egocentric (no spatial transformation) and altercentric (with spatial transformation) responses to differentiate the potential mechanisms driving either embodied or disembodied spatial transformation strategies during implicit altercentric perspective-taking.

Based on the alternative neurocognitive accounts, we explored multiple areas associated with the hMNS (IFG, IPL and PMv) and CCN (DLPFC, IFG and motor cortex; MC) but focused on the left IFG and bilateral DLPFC as critical regions of interest (ROIs) of the hMNS and CCN networks, respectively. If implicit altercentric perspective-taking is driven by spontaneous, *embodied* spatial transformation strategies supported by the hMNS, we hypothesized greater cortical activation in the left IFG but not in the left or right DLPFC for participants choosing an altercentric perspective than for those choosing an egocentric perspective. However, if implicit altercentric perspective-taking is accomplished via deliberate, *disembodied* cognitive control strategies supported by the CCN, we predicted greater cortical activation either singularly in the left or right DLPFC, or bilaterally, or possibly a co-activation in the IFG for participants choosing the altercentric perspective over the egocentric perspective. Subsequently, if the embodied hypothesis holds true, either comparable reaction times between the altercentric and egocentric responses or longer altercentric responses could be expected, depending on the extent of mitigation by the hMNS of any cognitive costs involved in the mental rotation process. If the disembodied hypothesis holds true, we expected that altercentric responses would result in longer reaction times than egocentric responses due to effortful spatial transposing and/or egocentric response inhibition strategies.

## Materials and methods

### Participants

A total of 120 students from the University of Tübingen who were either highly proficient or native-German speakers participated in this experiment for monetary compensation (8 Euros/hr). All participants had normal or corrected-to-normal vision and were required not to have a self-reported history of hearing, language, mental or neurological disorder. All participants provided written informed consent prior to the start of the experiment. Three participants had to be excluded from the analyses due to technical issues (missing triggers for one; noisy fNIRS data for two). Data from the remaining 117 participants (84 females, 33 males; mean age = 24.42 ± 3.99 years; age range: 18–37 years; 7 left-handed; 1 ambidextrous) were included in the analyses. The experimental procedure and method were carried out in accordance with the principles and guidelines of the Declaration of Helsinki, and the study protocol was approved by the local research ethics committees at the University Hospital Tübingen and Leibniz Institut für Wissensmedien.

### Paradigm design

**Figure 1:**
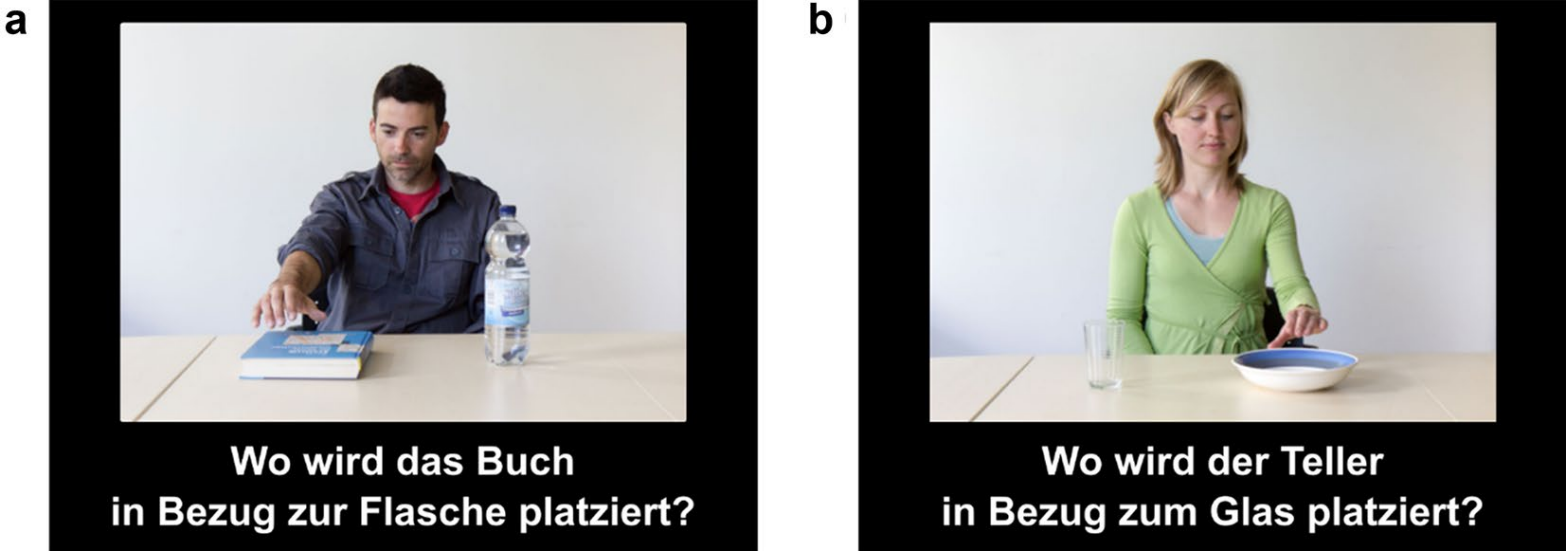
Examples of experimental stimuli. (**a**) The scene depicts a male agent reaching for a book, placed next to a bottle, with his right hand. (**b**) The scene depicts a female agent reaching for a plate, placed next to a glass, with her left hand. [Images in figure are licensed under CC BY 4.0].

The paradigm design consisted of picture stimuli of 24 similar scenes. Each scene showed an agent (front-facing the participant) seated at a table and reaching for one of two objects (book, bottle, glass or plate) placed side by side on the table with either the left or right hand (see Fig. 1). Two male and two female agents were each featured six times throughout the stimuli. Each of the six possible pairings of the four assorted objects (bottle-book, bottle-plate, bottle-glass, book-plate, book-glass, glass-plate) was presented four times throughout the experiment, such that each object in the pair would appear to the left and right of the participant twice. The agent’s right hand was seen reaching for the object to the left of the participant in half of the stimuli, while the left hand was seen reaching for the object to the right of the participant in the other half. Beneath each photograph, a question about the spatial relations between the objects was presented in German on the screen in the following structure, e.g., “Where is the book placed in relation to the bottle?” Participants were instructed to respond to the question with either “left” or “right” by pressing the corresponding arrow key with their dominant hand. No explicit instruction to take their own or the agent’s perspective was given. This deliberately restrictive set-up ensured that participants made spatial judgments implicitly from either their own (egocentric) or the agent’s (altercentric) perspective. To score the responses, if a participant responded with ‘left’ to a target object that was located to the left of the screen (aligned with the participant’s reference frame; e.g., Fig. 1a), that response was automatically coded as egocentric. However, if the participant responded with ‘right’ in that trial, that response was automatically coded as altercentric. In other words, responses congruent with the target object’s position on either side of the screen (participant’s reference frame) were coded as egocentric while incongruent responses were coded as altercentric.

### Test of spatial ability

To control for spatial abilities such as spatial visualization and mental rotation which can cause individual differences in transformation strategies involving forms and objects during virtual perspective-taking tasks^62,63^, a short version (Part A) of the Paper Folding Test^64^ (PFT) was administered. The PFT has been administered in previous fNIRS studies involving visualizations and has been shown to distinguish behavioral performance in relation to cortical activation^46,65^. Each of the ten items of the test consists of a probe and five response options. The probe provides stepwise depictions of how a square sheet of paper is folded (ranging from two to four folds) and punched with a hole in the last step (see example item in Supplementary Fig. S1 online). Participants were required to imagine the folding-punch-unfolding process and identify the option with the correct resulting punch configuration. Participants were given three minutes for the test. One point was awarded for each correct answer (maximum of 10 points).

### Experimental procedure

**Figure 2:**
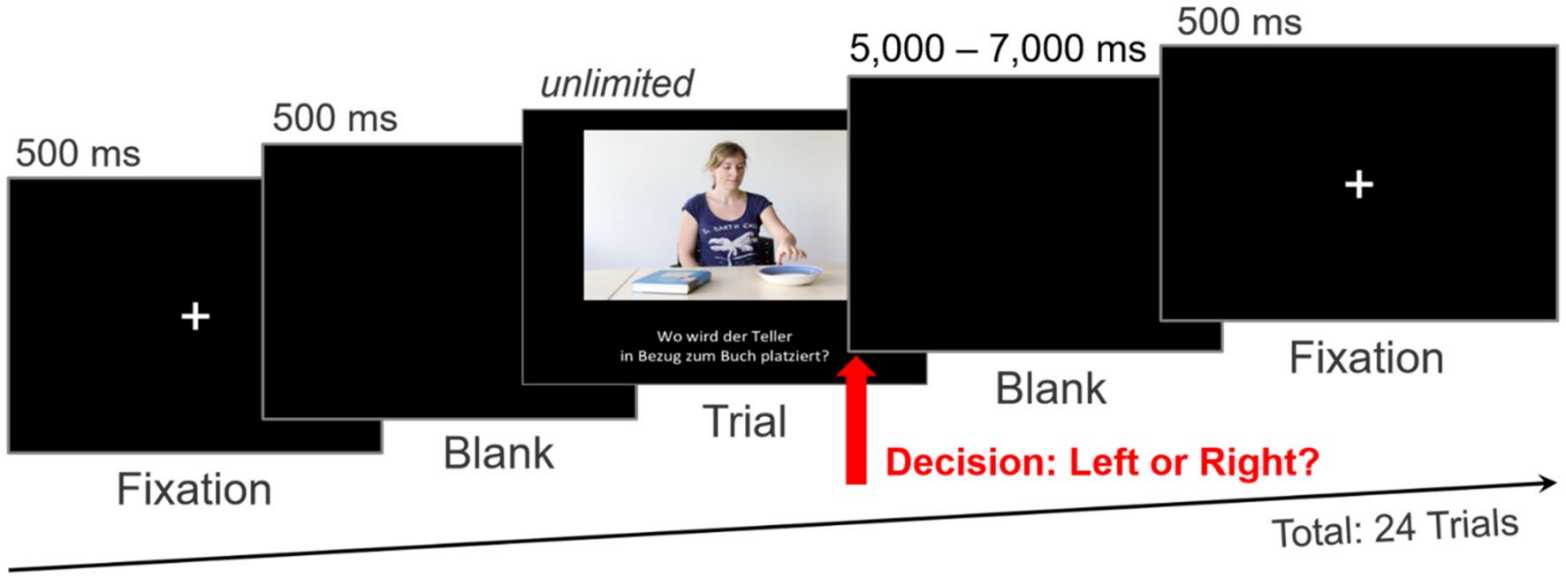
Illustration of trial presentation sequence including the inter trial interval. [Image in figure is licensed under CC BY 4.0].

Participants were individually tested in a sound-attenuated booth. After they provided signed informed consent and completed the PFT, the fNIRS probe sets were fastened to their heads (see next section for placement details). The stimuli were displayed on a 22-inch LCD monitor (Eizo Cooperation, Japan) which provided one of two minimal light sources (the other being the fNIRS device) in an otherwise darkened room. Participants’ distance to the screen was approximately 60 cm. The stimuli were presented with Presentation^®^ Version 16.3 (Neurobehavioral Systems Inc., Berkeley, CA, USA) in a randomized order. Each of the 24 trials began with a 500 ms fixation cross followed by a 500 ms blank black-screen and the subsequent onset of the picture stimuli with the corresponding question. The presentation duration for each stimulus was response-locked. Participants’ ‘left’ or ‘right’ keyboard response automatically triggered a black screen inter-trial interval with a duration jittered between 5000 and 7000 ms before the fixation cross for the next trial appeared. RT (in ms) was recorded as the difference between the point in time when the stimulus appeared and the point in time the participant gave a response. All participants completed a total of 24 trials. Figure 2 illustrates the presentation succession for each trial.

### Functional near-infrared spectroscopy

**Figure 3:**
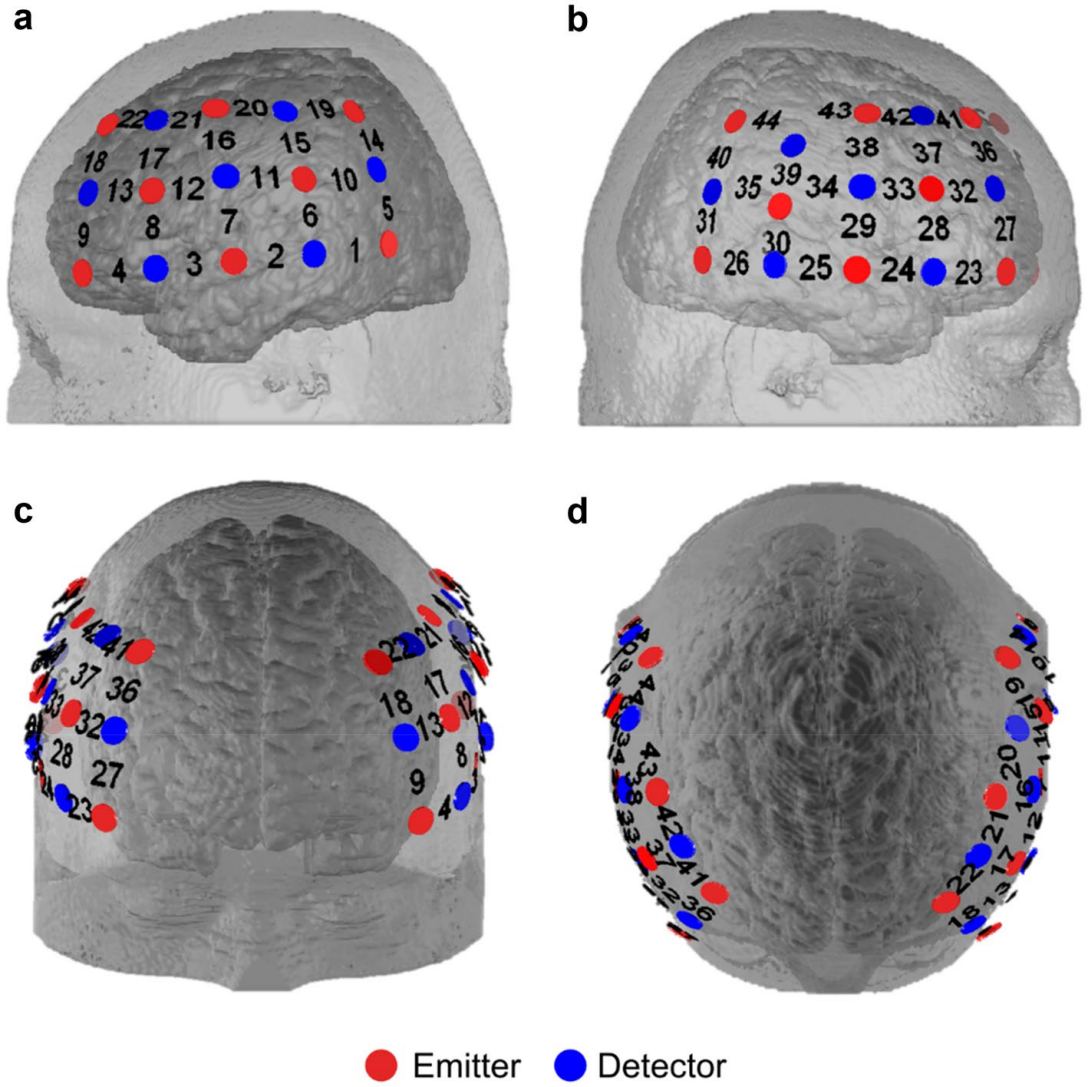
Schematic representation of emitters (red circles), detectors (blue circles) and resulting channels (numbers in black) in a symmetrical 3 × 5 placement configuration over both hemispheres. (**a**) Left hemisphere (channels 1–22). (**b**) Right hemisphere (channels 23–44). (**c**) Front view. (**d**) Top view.

**Figure 4:**
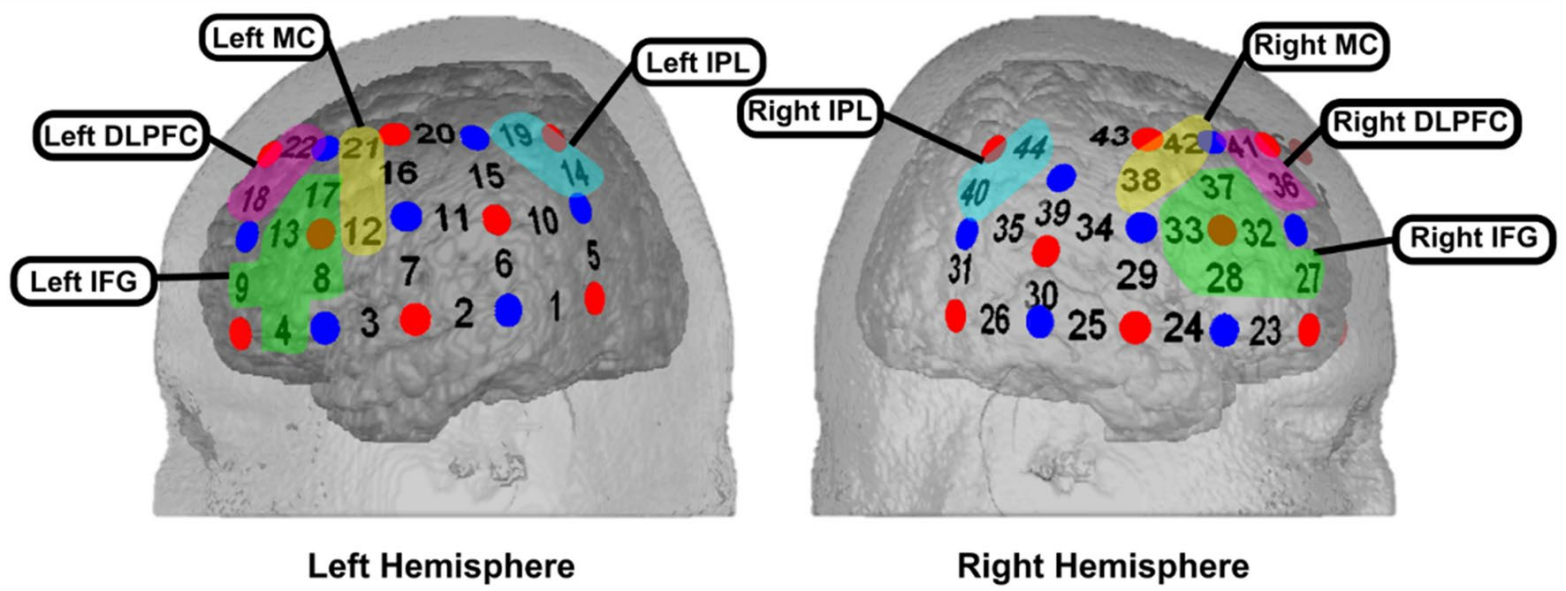
Channel assignments for defined regions of interest in left and right hemisphere probe sets.

fNIRS is an optical imaging technique that measures concentration changes in the levels of cortical oxygenated (O_2_Hb) and deoxygenated (HHb) hemoglobin. This technique was selected because it has relatively low sensitivity to motion artifacts, imposes few constraints on body posture and therefore allows participants to be seated upright, mirroring the position of the agent^66–68^. fNIRS data was collected with the multi-channel, continuous-wave ETG-4000 Optical Topography System (Hitachi Medical Co., Japan) at a sampling rate of 10 Hz. This unit utilizes avalanche laser diodes at two wavelengths (695 ± 20 and 830 ± 20 nm) with 4.0.2 mW at each optode per wavelength^69^. Two 3 × 5 probe sets, each comprising eight light emitters and seven detectors (yielding 22 channels distributed over each hemisphere at a fixed inter-optode distance of 30 mm), were attached to either side of each participant’s head in a standardized fashion according to the international 10–20 system for electrode placement^70^. The left probe set was oriented such that channels 2 and 4 were centered on T3 and F7 respectively, while the right probe set was symmetrically oriented such that channels 25 and 23 were centered on T4 and F8 respectively (refer to Fig. 3). This procedure provided consistent placement of probe sets over the bilateral IFG, bilateral DLPFC and parts of the bilateral parietal cortices across participants. The channel locations were determined via a virtual, probabilistic registration process based on the standard Colin 27 template^71–74^ and their corresponding anatomical locations (Fig. 4) were estimated according to the automated anatomical labeling (AAL) atlas in the Statistical Parametric Mapping (SPM) package^75^.

### Data analysis

#### Behavioral data

Participants who had proportionally more egocentric-labeled trials than altercentric-labeled trials overall were categorized into the egocentric group (n = 73), and vice versa for the altercentric group (n = 44). Trials with RTs deviating more than 3 standard deviations (*SD*) from a participant’s overall mean were excluded from further analyses (2.17% of observations from the egocentric group, 1.52% from the altercentric group). The two groups did not differ in proportion of eliminated trials, *χ*^*2*^(1) = 1.493, *p* = 0.222.

#### fNIRS data preprocessing

The collected fNIRS data was preprocessed in MATLAB R2020a (MathWorks Inc., Natick, USA) using customized scripts as in previous studies from our group^76,77^. After the absorbed NIRS light was converted into O_2_Hb and HHb concentration changes by employing the modified Beer-Lambert law, a band-pass filter (0.001–0.2 Hz) was first applied to the hemodynamic signals to remove high and low frequency noise. Next, the correlation-based signal improvement (CBSI) algorithm by Cui et al.^78^ was applied to reduce low amplitude motion artifacts and other non-evoked systemic influences, whilst merging O_2_Hb and HHb into one signal to assess functional activation from both signals simultaneously^79,80^. An independent component analysis (ICA) was then performed for certain participants to filter out distinct systemic confounds such as teeth-clenching artifacts (approx. 17% of sample). A principal component analysis (PCA) was performed for participants whose CBSI-corrected signal presented with too many high-amplitude teeth-clenching artifacts than recommended for removal with the ICA (approx. 25% of sample)^81^. At this point, through stringent comparison of amplitude plots between pre- and post-component removal, signals necessitating the removal of more than 10% of components to eradicate striking deviance were excluded from the dataset for further analyses (two participants). After the removal of selected components in signals corrected by the ICA or PCA, individual channels whose amplitude still displayed substantial deviation from the average maxima of other channels were interpolated with neighboring channels (approx. 6% of ICA-/PCA-corrected sample; average of 4 channels). Likewise, highly deviant channels in signals not previously subjected to ICA or PCA correction were also interpolated (approx. 8% of sample; average of 2 channels). In our sample, no more than 9 channels at a time had to be interpolated per subject (on average 3.5 interpolations across affected subjects). 48 total interpolations (of 24 out of 44 channels) were evenly distributed between the left and right probe sets, *χ*^*2*^(1) = 0.084, *p* = 0.772, and between the critical and non-critical ROIs, *χ*^*2*^(1) = 0.146, *p* = 0.910 (refer to the next section for the classification of ROIs). A second bandpass filter (0.01–0.2 Hz) was applied before a global signal reduction was performed by means of a spatial Gaussian PCA-based kernel filter^82^ with a standard deviation of σ = 40. Finally, the pre-processed fNIRS signal for each participant was z-standardized for better comparison among participants before a GLM was fit. The regressor was obtained by convolving the event onsets of each of the 24 trials with the canonical hemodynamic response function (HRF) using a peak time of 6.5 s (HRFs for sensorimotor tasks peak between 5 and 10 s, with a latency lag of about 1.5 s^83–86^). Single-channel beta values were derived per participant for further statistical analyses.

### Statistical analysis

All statistical analyses of behavioral and neurophysiological data were conducted using IBM SPSS Statistics Version 25 (Armonk, NY, USA) and R^87^ Version 4.0.3. For all analyses, the criterion for significance was *p* < 0.05. In light of our exploratory study, reported statistical calculations including *p*-values and confidence intervals pertaining to cortical activation are not confirmatory and are to be interpreted with caution. Multiple comparisons are uncorrected and reported results are for descriptive purposes only with no claims for generalizability^88^.

#### Primary analysis

Cortical activation in the left IFG was analyzed as the critical ROI of the hMNS while cortical activation in the left and right DLPFC was analyzed as the critical ROIs of the CCN. Changes in cortical activation in these three critical and five remaining ROIs were analyzed with the single between-subjects factor perspective (egocentric versus altercentric) using separate, independent t-tests (two-tailed). RT differences between the egocentric (n = 73) and altercentric (n = 44) groups were likewise analyzed with a two-tailed independent t-test. Cohen’s *d* values are reported as an estimate of effect size for the t-tests.

#### Secondary analysis

Contrary to the single responses Tversky and Hard’s^1^ paradigm yielded per participant, our multiple-trial paradigm revealed perspective-switching behavior between trials for one-fifth of the participants, thereby introducing complexities to group categorization. To allow for finer-grained distinctions between response strategies at the neurophysiological and behavioral levels, we added consistency (consistent versus inconsistent) as a second between-subjects factor. We anticipated that an overall consistent response pattern (e.g., applying a fixed ‘rule’ throughout the trials) in relation to cortical activation in the various networks would suggest an automatized processing strategy. Conversely, observed inconsistencies in the response pattern, in relation to cortical activation, would indicate a more effortful and deliberate processing strategy. Table 1 summarizes the adjusted hypotheses incorporating consistency.

**Table 1:** Comparison of strategies and ROIs between embodied and disembodied accounts.

	Embodied account	Disembodied account
**Cognitive mechanism**	Spontaneous & automatic transformation	Deliberate & effortful transformation
**Neural network**	Human mirror neuron system (hMNS)	Cognitive control network (CCN)
**Possible transformation strategies**	Mental self-rotation; motor simulation of observed action from altercentric perspective	Inhibition of interference from egocentric perspective; spatial transposing of left/right
**Regions of interest** ^§^Critical	Left IFG^**§**^, right IFG, left IPL, right IPL, left MC, right MC	Left DLPFC^**§**^, right DLPFC^**§**^, left IFG, right IFG, left MC, right MC
**Expected patterns of cortical activation in ROIs**	Altercentric > Egocentric Altercentric-C > Egocentric-C Altercentric-C > Egocentric-IC Altercentric-C > Altercentric-IC	Altercentric > Egocentric Altercentric-IC > Egocentric-IC Altercentric-IC > Egocentric-C Altercentric-IC > Altercentric-C
**Possible patterns of reaction times**	Altercentric ≥ Egocentric Altercentric-C ≥ Egocentric-C Altercentric-IC ≥ Egocentric-IC Altercentric-IC > Egocentric-C Altercentric-IC > Altercentric-C	Altercentric > Egocentric Altercentric-C > Egocentric-C Altercentric-IC > Egocentric-IC Altercentric-IC > Egocentric-C Altercentric-IC > Altercentric-C

*C* consistent, *IC* inconsistent, *ROI* region of interest.

Participants were re-categorized into four subgroups based on the self-selecting nature of the implicit paradigm. Those who maintained the same perspective on at least 23 out of 24 trials (5% cut-off; one trial buffer) were assigned to either the egocentric-consistent (n = 64) or altercentric-consistent (n = 31) subgroups. Participants who switched between perspectives more than once (22 or lesser trials with the same perspective) were assigned to either the egocentric-inconsistent (n = 9) or altercentric-inconsistent (n = 13) subgroups.

Cortical activation in the three critical ROIs were analyzed with separate two-way Analyses of Variance (ANOVAs) in a 2 × 2 factorial design with the between-subjects factors perspective (egocentric versus altercentric) and consistency (consistent versus inconsistent). All homogeneity of variance assumptions were met. Partial eta squared (*η*^*2*^_*p*_) values are reported as an estimate of effect size for the ANOVAs. Type III Sum of Squares was used to cater for the varying subgroup sizes by adjusting the sum of squares based on the assumption of a balanced design^89^.

RT differences among the four subgroups were likewise analyzed with a two-way ANOVA with the factors perspective and consistency. To further analyze the impact of perspective on reaction time with respect to trial-by-trial perspective-switching behavior by participants in the inconsistent groups, an RT analysis was performed at the individual trial response level. Using a linear mixed-effects model, generated with the lmer function in the lme4 R package^90^ and fit by a maximum likelihood technique, the between-subjects factor group (egocentric-inconsistent versus altercentric-inconsistent) and within-subjects factor response (implied perspective translated from left or right responses, i.e., egocentric-response versus altercentric-response) were set as fixed effects. The random effects included intercepts for trial and a by-subject random slope for the interaction between response and subjects. Effect sizes for the linear mixed-effects model were calculated using the MuMIn R package^91,92^. They are reported in the form of a marginal *R*^*2*^_*m*_ value, representing the variance explained by the fixed effects, and the conditional *R*^*2*^_*c*_ value, representing variance explained by both fixed and random effects. Post-hoc pairwise comparisons were computed with a Bonferroni correction.

#### Exploratory analysis

To potentially bolster any effects from the primary and secondary analyses, separate two-way ANOVAs with the factors perspective and consistency on cortical activation in five additional ROIs were run (right IFG, left IPL, right IPL, left MC and right MC). All five ROIs were associated with the hMNS while three were simultaneously associated with the CCN (right IFG, left MC and right MC).

To further explore the influence of spatial abilities on potential cognitive perspective-taking strategies either driven by the hMNS or CCN, PFT scores were correlated with cortical activation in all critical and additional ROIs. Pearson correlation coefficients, *r*, are reported.

## Results

### Sample characteristics

Participants did not differ in sex, handedness, age or spatial abilities (PFT scores) between the egocentric and altercentric groups. Similarly, there were no differences in sex, handedness, and age among the four subgroups (Table 2). However, consistent participants had significantly higher PFT scores than inconsistent participants, *F*(1,112) = 4.814, *MSE* = 3.559, *p* = 0.030, *η*^*2*^_*p*_ = 0.041. There was neither a main effect for perspective nor any interaction between perspective and consistency for spatial abilities (both *F*s < 1).

**Table 2:** Descriptive statistics of participant subgroups.

	Egocentric (n = 73)		Altercentric (n = 44)	
	Consistent (n = 64)	Inconsistent (n = 9)	Consistent (n = 31)	Inconsistent (n = 13)
**Sex**				
Females (n = 84)	45	6	24	9
Males (n = 33)	19	3	7	4
**Handedness**				
Ambidextrous	–	–	–	1
Left-handed	5	–	2	–
Right-handed	59	9	28	12
**Age**	24.73 (4.05)	24.22 (4.15)	24.29 (3.87)	23.31 (4.09)
**Spatial ability** (PFT score)	7.69 (1.81)	6.44 (2.13)	7.23 (2.05)	6.46 (1.71)

*PFT* paper folding test. Standard deviations of means for age and PFT score in parentheses.

### Primary analysis

#### Impact of perspective-taking on cortical activation

Results from the independent t-tests showed no significant differences in cortical activation between the egocentric and altercentric groups in either the critical ROIs or exploratory ROIs (all p > 0.05; see Table 3 for cortical activation means and Table 4 for t-test statistics).

**Table 3:** Mean cortical activation (in beta values) in critical and exploratory ROIs as a function of perspective.

ROI	Egocentric (n = 73)	Altercentric (n = 44)
**Critical ROIs**		
Left IFG	3.240 (10.841)	4.494 (15.579)
Left DLPFC	0.616 (18.008)	0.755 (17.368)
Right DLPFC	1.588 (16.086)	− 0.757 (16.347)
**Exploratory ROIs**		
Right IFG	3.038 (9.402)	3.108 (13.340)
Left IPL	− 8.231 (18.971)	− 5.477 (19.988)
Right IPL	− 4.030 (20.061)	− 2.539 (21.549)
Left MC	10.330 (13.448)	10.253 (21.560)
Right MC	− 0.883 (15.623)	3.276(22.051)

Standard deviations of means in parentheses.

**Table 4:** Summary of t-test statistics in critical and exploratory ROIs as a function of perspective.

ROI	*t*	*p*	*d*	95% CI
**Critical ROIs**				
Left IFG	− 0.513	0.609	− 0.098	[− 6.101, 3.592]
Left DLPFC	− 0.041	0.967	− 0.008	[− 6.858, 6.579]
Right DLPFC	0.759	0.449	0.145	[− 3.773, 8.464]
**Additional ROIs**				
Right IFG	− 0.033	0.974	− 0.006	[− 4.244, 4.104]
Left IPL	− 0.745	0.458	− 0.142	[− 10.072, 4.565]
Right IPL	− 0.379	0.706	− 0.072	[− 9.290, 6.308]
Left MC	0.024	0.981	0.005	[− 6.329, 6.481]
Right MC	− 1.191	0.236	− 0.227	[− 11.075, 2.757]

*CI* confidence interval.

### Impact of perspective-taking on reaction time

The independent t-test on RT revealed that participants in the altercentric group took significantly longer to respond across the trials than those in the egocentric group (*t* = 2.523, *p* = 0.014, *d* = 0.471; see Table 5 for RT group means).

**Table 5:** Mean reaction time as a function of perspective.

	Egocentric (n = 73)	Altercentric (n = 44)
**Reaction time** (in ms)	2869.47 (1997.02)	3433.21 (1618.11)

Standard deviations of means in parentheses.

### Secondary analysis

#### Impact of perspective and consistency on cortical activation

**Figure 5:**
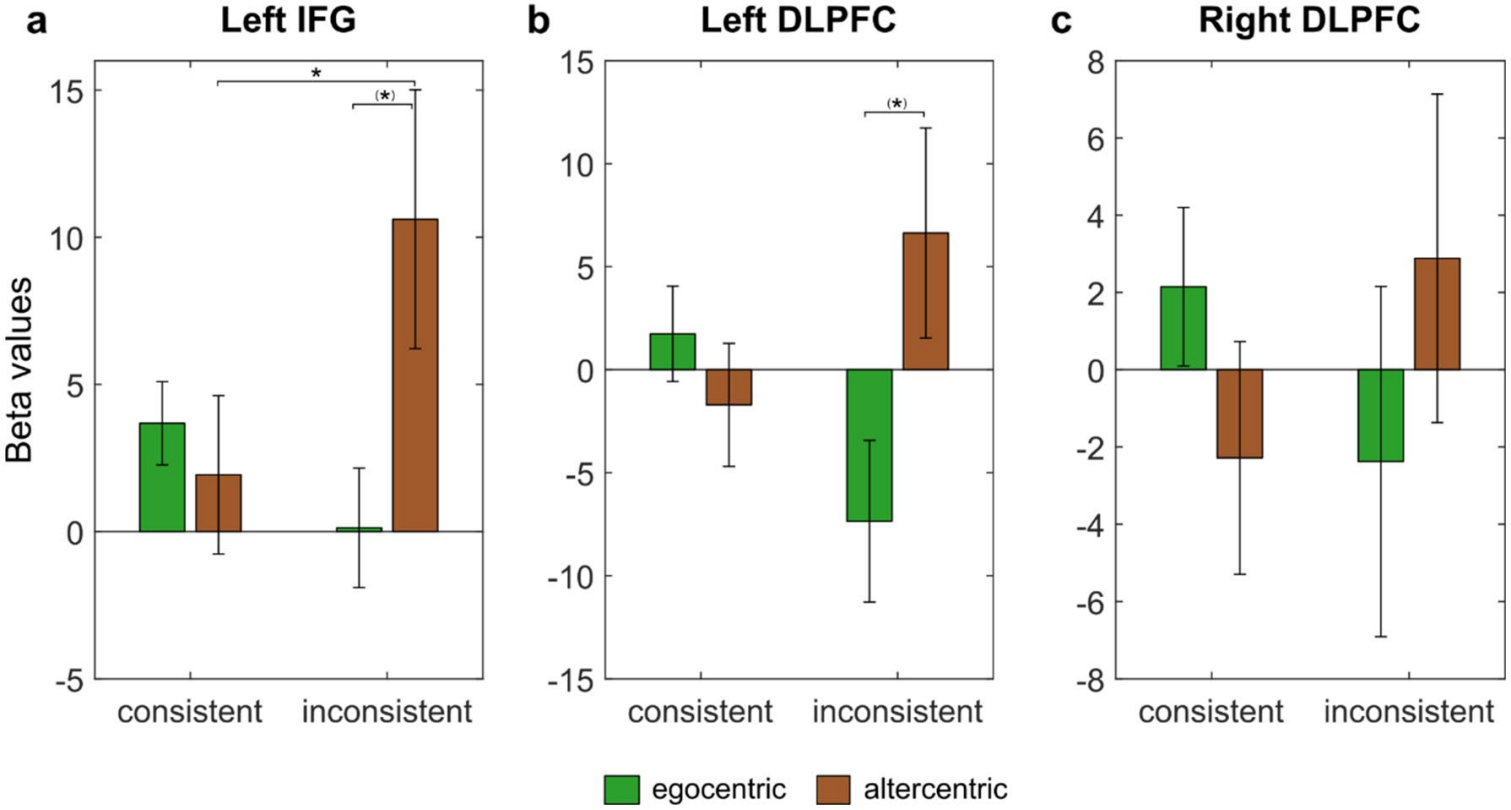
Cortical activation for interaction between factors perspective and consistency in (**a**) Left IFG, (**b**) Left DLPFC, and (**c**) Right DLPFC. *p < 0.05, ^(^*^)^*p* < 0.10. Error bars represent standard errors.

**Figure 6:**
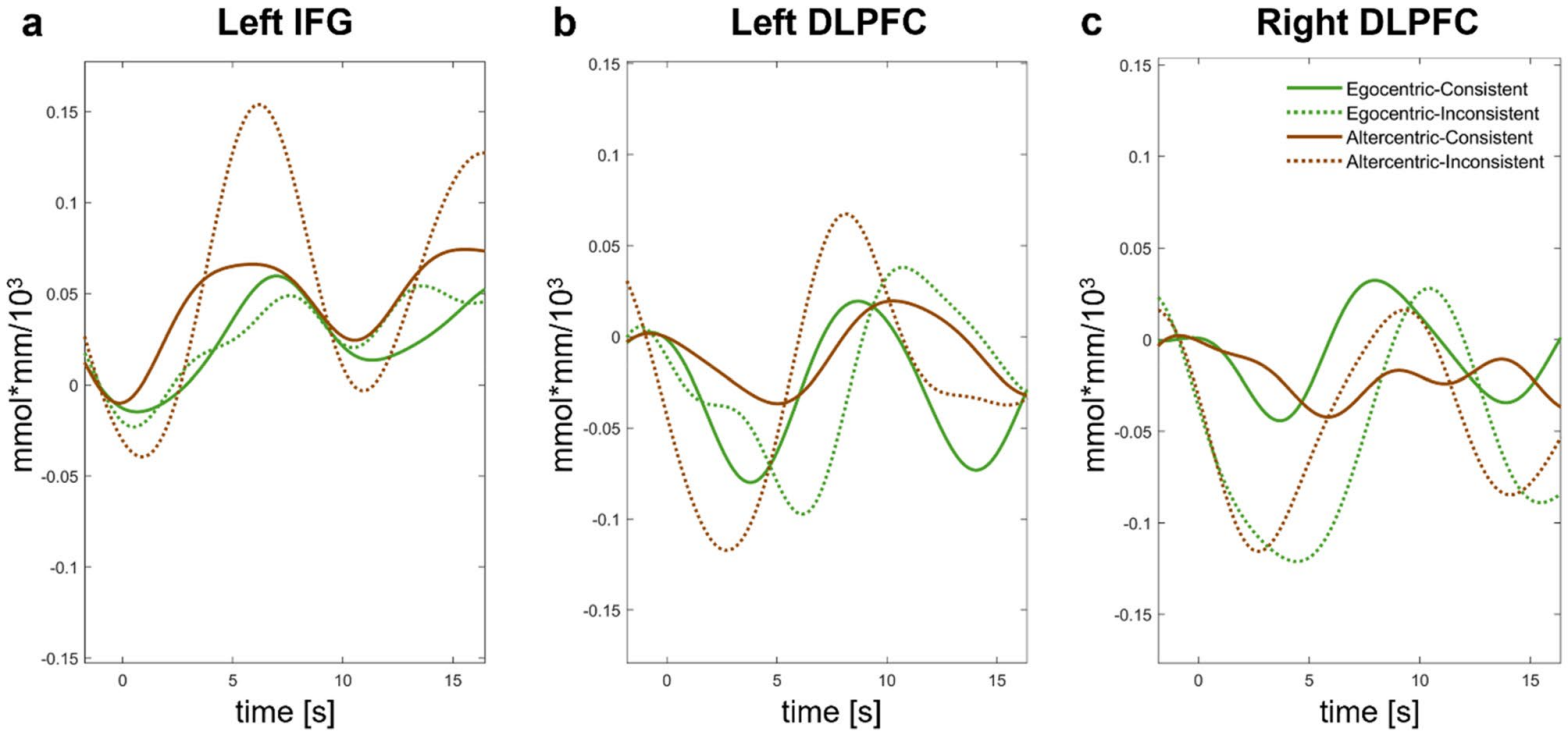
Event related averaged hemodynamic time course plots for the subgroups in (**a**) Left IFG, (**b**) Left DLPFC, and (**c**) Right DLPFC.

None of the three two-way ANOVAs with the factors perspective (egocentric versus altercentric) and consistency (consistent versus inconsistent) on cortical activation in the three critical ROIs yielded a main effect for perspective or for consistency (all *p* > 0.05). However, there was a significant interaction between perspective and consistency on cortical activation in both the left IFG, *F*(1,113) = 3.958, *MSE* = 634.249, *p* = 0.049, *η*^*2*^_*p*_ = 0.034, and left DLPFC, *F*(1,113) = 4.158, *MSE* = 1289.080, *p* = 0.044, *η*^*2*^_*p*_ = 0.035, but not the right DLPFC, *F*(1,113) = 1.513, *MSE* = 398.006, *p* = 0.221, *η*^*2*^_*p*_ = 0.013 (Fig. 5; see Fig. 6 for hemodynamic plots).

Within the left IFG, uncorrected post-hoc pairwise comparisons showed significantly greater cortical activation for the altercentric-inconsistent subgroup than for the altercentric-consistent subgroup (*p* = 0.045). Cortical activation between the altercentric-inconsistent and egocentric-inconsistent subgroups were not significantly different (*p* = 0.060). However, within the left DLPFC, no substantial differences in cortical activation between the altercentric subgroups (*p* = 0.165), and between the altercentric-inconsistent and egocentric-inconsistent subgroups, were captured (*p* = 0.070). Table 6 reports the cortical activation means in the critical ROIs for the four subgroups.

**Table 6:** Mean cortical activation (in beta values) in critical ROIs as a function of perspective and consistency.

ROI	Egocentric (n = 73)		Altercentric (n = 44)	
	Consistent (n = 64)	Inconsistent (n = 9)	Consistent (n = 31)	Inconsistent (n = 13)
**Left IFG**	3.677 (11.316)	0.129 (6.082)	1.928 (14.976)	10.612 (15.856)
**Left DLPFC**	1.737 (18.511)	− 7.357 (11.767)	− 1.711 (16.609)	6.635 (18.387)
**Right DLPFC**	2.146 (16.423)	− 2.377 (13.591)	− 2.284 (16.757)	2.883 (15.337)

Standard deviations of means in parentheses.

#### Impact of perspective and consistency on reaction time

**Figure 7:**
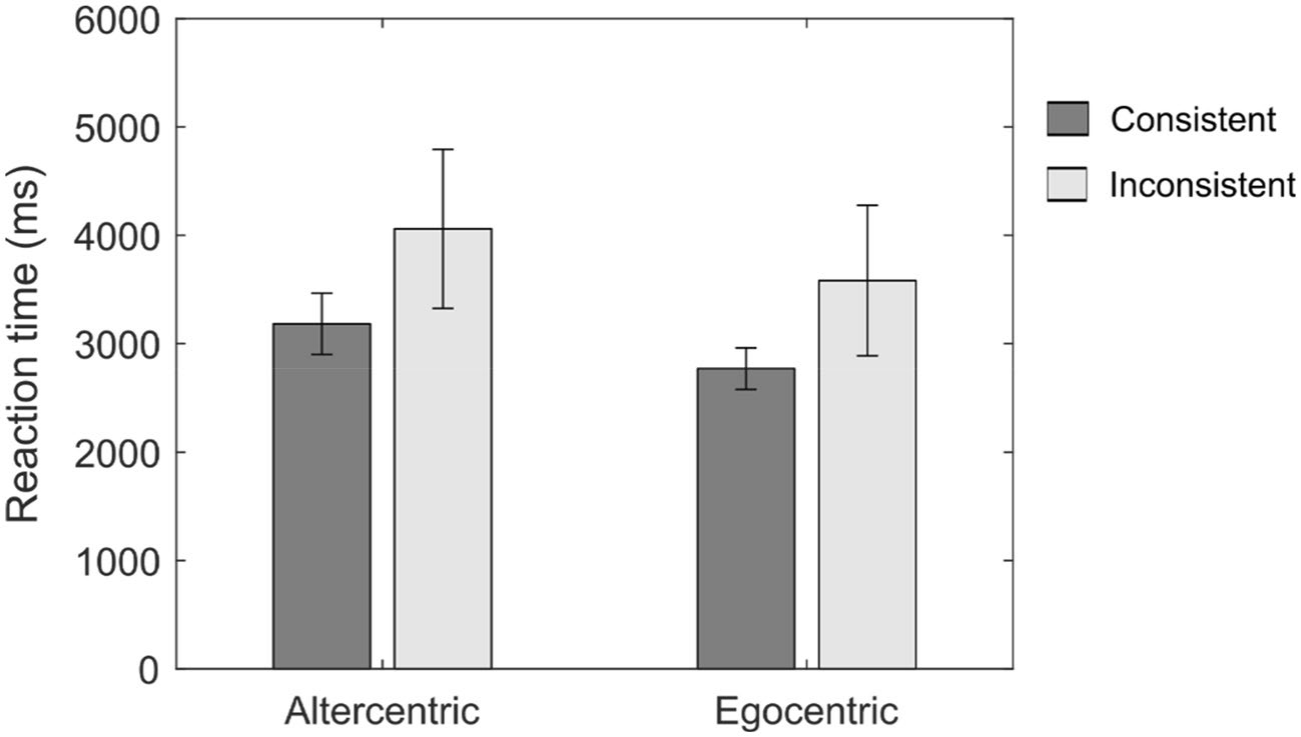
Mean reaction time as a function of perspective and consistency in subgroups. Error bars represent standard errors.

The two-way ANOVA on RT by perspective (egocentric versus altercentric) and consistency (consistent versus inconsistent) showed a main effect for consistency, *F*(1,113) = 6.405, *MSE* = 7,049,471.78, *p* = 0.013, *η*^*2*^_*p*_ = 0.054, but not for perspective anymore [*F*(1,113) = 3.085, *MSE* = 3,395,276.08, *p* = 0.082, *η*^*2*^_*p*_ = 0.027]. There was no significant interaction between the two factors either (*F* < 1). Participants who were inconsistent in maintaining one implicit perspective throughout the trials (i.e., switched perspectives twice or more times between trials) took overall significantly longer to respond than participants who were consistent (Fig. 7).

#### Trial-based response and switches on reaction time

**Figure 8:**
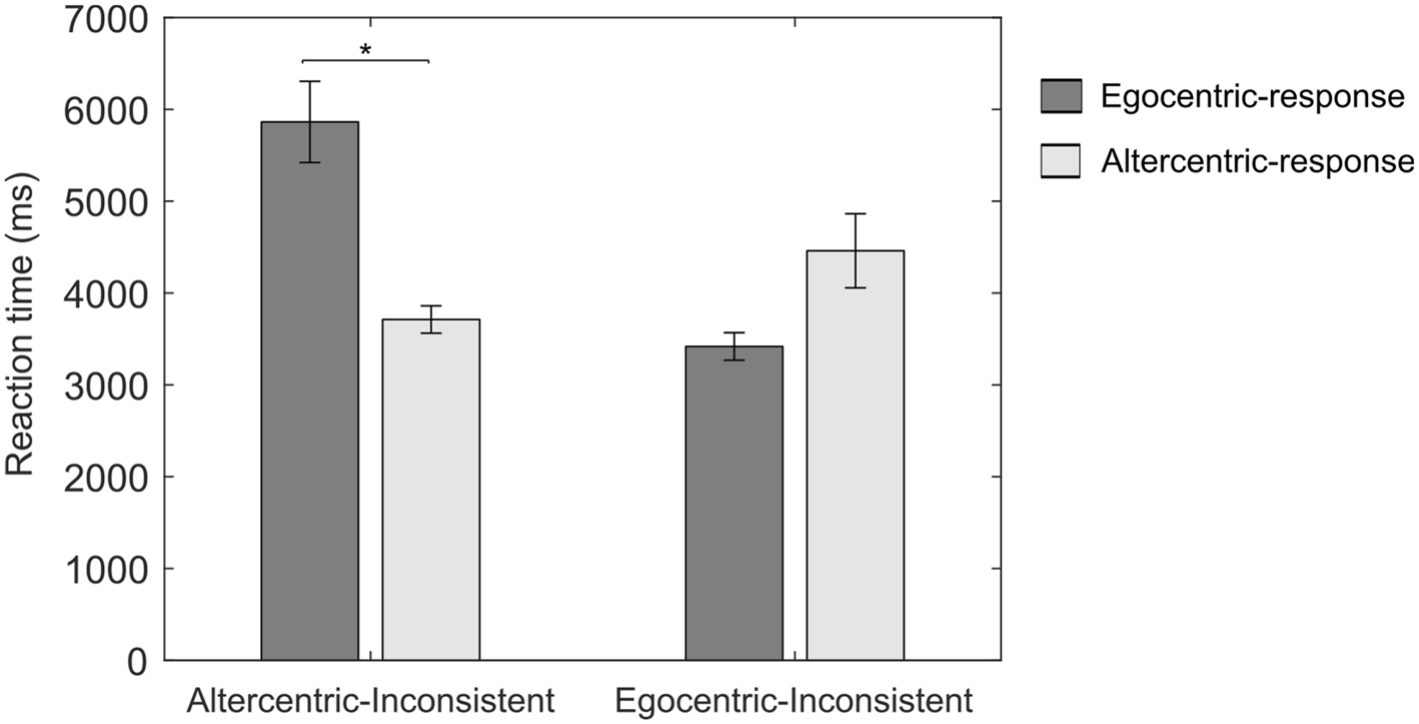
Mean reaction time as a function of group and response in inconsistent subgroups. Error bars represent standard errors.

Results from the linear mixed-effects model for RTs by inconsistent participants showed that the within-subjects factor response (egocentric-response versus altercentric-response; *t* = 3.257, *p* = 0.004) but not the between-subjects factor group (egocentric-inconsistent versus altercentric-inconsistent; *t* = 0.998, *p* = 0.327) could significantly predict the variance in RT (*R*^*2*^_*m*_ = 0.038). This was qualified by a significant interaction between group and response on log RT (*t* = − 3.976, *p* = 0.001,* R*^*2*^_*c*_ = 0.579; see Supplementary Table S1 for summary of output). Post-hoc pairwise comparisons revealed that participants within the altercentric-inconsistent subgroup were significantly quicker on the altercentric responses than egocentric responses (*p* = 0.031; Fig. 8).

### Exploratory analysis

**Figure 9:**
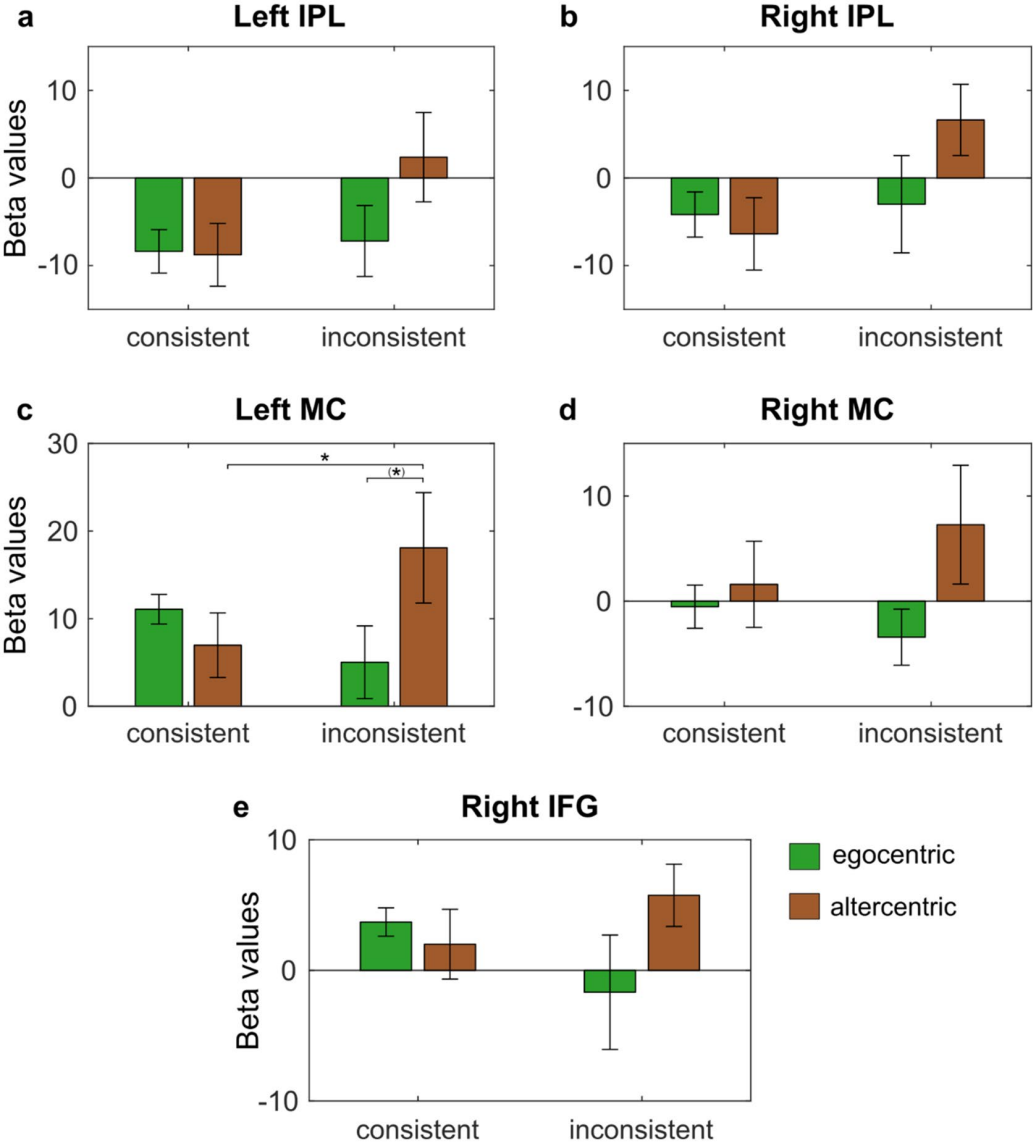
Cortical activation for interaction between factors perspective and consistency in (**a**) Left IPL, (**b**) Right IPL, (**c**) Left MC, (**d**) Right MC, and (**e**) Right IFG. *p < 0.05, ^(^*^)^*p* < 0.10. Error bars represent standard errors.

**Figure 10:**
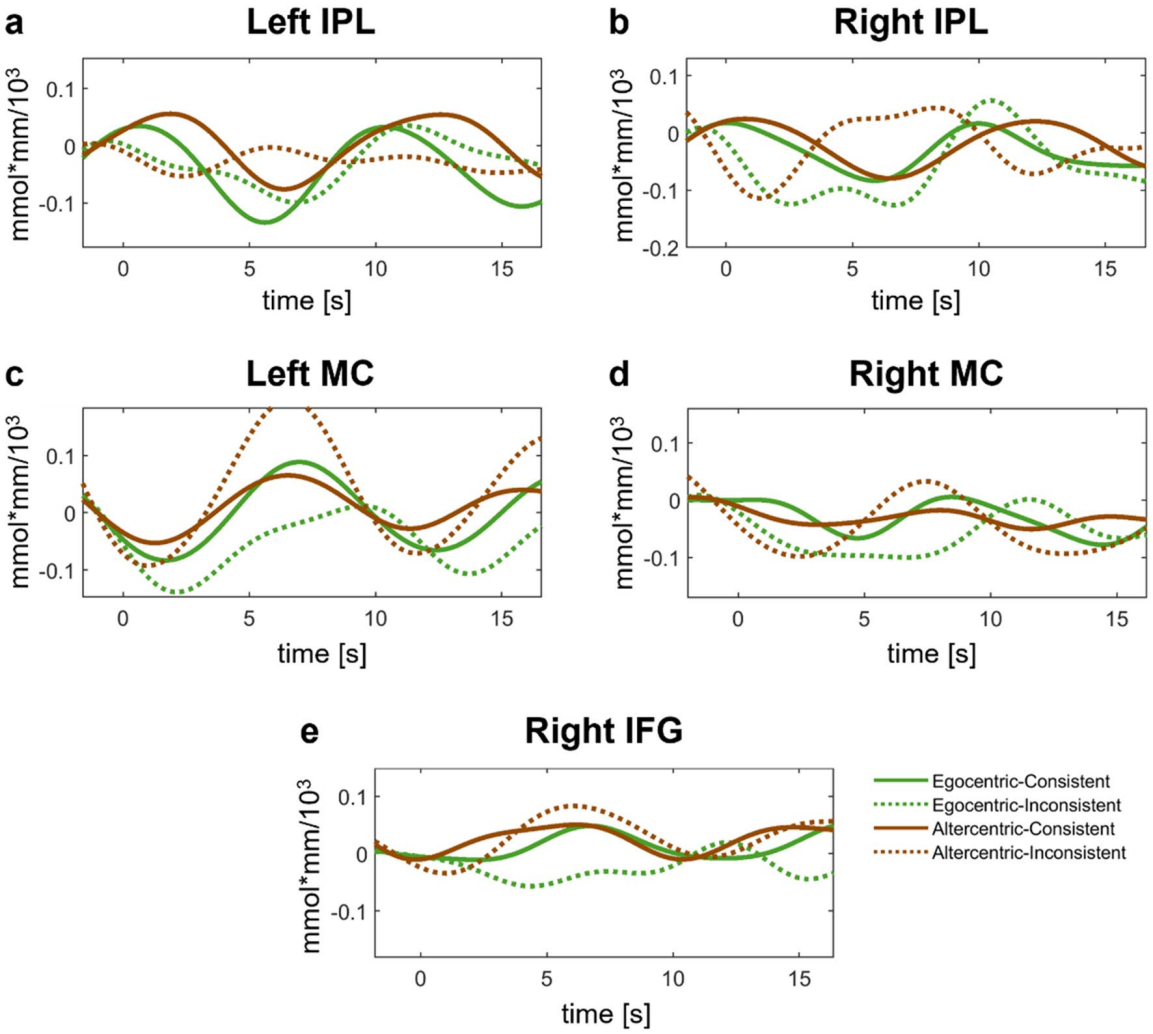
Event related averaged hemodynamic time course plots for the subgroups in (**a**) Left IPL, (**b**) Right IPL, (**c**) Left MC, (**d**) Right MC, and (**e**) Right IFG.

The two-way ANOVAs showed no main effects for the factors perspective and consistency in any of the five additional exploratory ROIs (all *p* > 0.05). There was only a significant interaction between the factors in the left MC, *F*(1,113) = 4.468, *MSE* = 1249.006, *p* = 0.037, *η*^*2*^_*p*_ = 0.038 (Fig. 9; see Fig. 10 for hemodynamic plots).Within the left MC, uncorrected post-hoc pairwise comparisons indicated significantly greater cortical activation for the altercentric-inconsistent subgroup than for the altercentric-consistent subgroup (*p* = 0.043). The cortical activation difference between the altercentric-inconsistent and egocentric-inconsistent subgroups did not meet significance (*p* = 0.076). Table 7 reports the cortical activation means in the exploratory ROIs and Table 8 presents the ANOVA statistics in all critical and exploratory ROIs.

**Table 7:** Mean cortical activation in exploratory ROIs as a function of perspective and consistency.

ROI	Egocentric (n = 73)		Altercentric (n = 44)	
	Consistent (n = 64)	Inconsistent (n = 9)	Consistent (n = 31)	Inconsistent (n = 13)
**Right IFG**	3.701 (8.689)	− 1.676 (13.134)	2.003 (14.873)	5.743 (8.603)
**Left IPL**	− 8.377 (19.809)	− 7.195 (12.149)	− 8.769 (19.986)	2.372 (18.390)
**Right IPL**	− 4.175 (20.603)	− 2.996 (16.661)	− 6.381 (22.981)	6.623 (14.650)
**Left MC**	11.075 (13.508)	5.026 (12.439)	6.969 (20.542)	18.085 (22.725)
**Right MC**	− 0.526 (16.425)	− 3.426 (7.998)	1.599 (22.827)	7.274 (20.376)

Standard deviations of means in parentheses.

**Table 8:** Summary of ANOVA statistics in critical and exploratory ROIs as a function of perspective and consistency (n = 117).

ROI	*F*	*MSE*	*P*	*η*^*2*^_*p*_
**Critical ROIs**				
Left IFG	3.958	634.249	0.049*	0.034
Left DLPFC	4.158	1289.080	0.044*	0.035
Right DLPFC	1.513	398.006	0.221	0.013
**Exploratory ROIs**				
Right IFG	2.915	352.338	0.091	0.025
Left IPL	1.133	420.489	0.289	0.010
Right IPL	1.413	592.685	0.237	0.012
Left MC	4.468	1249.006	0.037*	0.038
Right MC	0.924	311.720	0.338	0.008

*p < 0.05. The correlation analysis revealed no significant relationship between PFT scores and cortical activation in any of the eight critical and additional ROIs (all *p* > 0.05; see Table 9 for correlation matrix of variables).

**Table 9:** Pearson correlation coefficients (*r*) for the relation of PFT score and cortical activation in ROIs (n = 117).

ROI	PFT Score	
	*r*	*p*
**Critical ROIs**		
Left IFG	− 0.033	0.726
Left DLPFC	− 0.008	0.930
Right DLPFC	0.003	0.974
**Exploratory ROIs**		
Right IFG	− 0.002	0.987
Left IPL	0.037	0.693
Right IPL	0.066	0.481
Left MC	0.034	0.713
Right MC	0.054	0.563

## Discussion

The present fNIRS study investigated the influence of action observation on implicit SPT by exploring whether spatial transformation strategies underlying implicit altercentric perspective-taking—associated with either an embodied or disembodied neurocognitive account—could be predicted on the basis of cortical activation. Using an adapted left–right spatial judgment task^1^ that was designed to elicit unprompted perspective-taking in the presence of agentive action cues, we attempted a multi-step exploratory approach to distinguish the involvement of the hMNS and CCN (respective networks of the two accounts) in driving egocentric and altercentric perspective-taking.

In contrast to an expected 50–50% distribution of egocentric and altercentric responses, a majority of our participants (62%) implicitly selected an egocentric perspective on most trials while the remaining participants (38%) predominantly chose the altercentric perspective. This leading preference for making spatial judgments implicitly from the egocentric reference frame (no spatial transformation necessary) was reflected in significantly slower RTs from participants responding from an altercentric reference frame (spatial transformation necessary). However, as cortical activation in the altercentric group was not found to differ from that of the egocentric group in either the critical ROIs associated with the hMNS and CCN (left IFG, left DLPFC, and right DLPFC) or the remaining ROIs (right IFG, left IPL, right IPL, left MC, and right MC), these primary analyses were not adequate to differentiate between spatial transformation strategies at the neurophysiological level and thereby validate either the embodied or disembodied account.

Introducing ‘consistency’ as an additional between-subjects factor in the secondary analyses provided some insight into the plausible involvement of the left IFG and left DLPFC during implicit SPT. Participants who predominantly selected the altercentric reference frame throughout the trials but were overall inconsistent in their decisions (altercentric-inconsistent) activated the left IFG more greatly than those who were overall consistent (altercentric-consistent). In other words, being unsure about undertaking a spatial transformation strategy, rather than sure about that selection, results in greater use of the left IFG. Although only marginally significant, cortical activation patterns suggest that being irregular about performing a spatial transformation on most trials (altercentric-inconsistent) compared to being irregular about maintaining a non-transformation strategy on most trials (egocentric-inconsistent) could lead to greater activation in the left IFG and left DLPFC (Fig. 5).

In further exploratory analyses, only similar patterns of behavior by the altercentric-inconsistent subgroup as above were found in the left MC: The altercentric-inconsistent subgroup had significantly greater cortical activation than the altercentric-consistent subgroup, and marginally greater activation than the egocentric-inconsistent subgroup. Notably, there was an observed lack of significant cortical activation in the IPL. Taken together, these results seem to speak against a spontaneous and automatic processing strategy when inconsistently making implicit spatial judgments from an altercentric reference frame in our particular paradigm. Rather, they suggest effortful processing, in which inconsistency in deliberate decision-making, only in combination with perspective, might play a modulating role in the propensity for potential processing strategies. This is then potentially governed by the left IFG, left DLPFC, and left MC, which are defining areas of the CCN.

In our interpretation, the overall pattern of observations in these subgroups seems to suggest a more plausible engagement of the CCN, as opposed to the hMNS, as one of the underlying neural networks in this task. Although inconclusive, they allude support of slightly more features under the disembodied account as compared to the embodied account. We speculate that the cognitive behavior of inconsistently making spatial judgments from the altercentric reference frame involves lapses in the ability to maintain a deliberate strategy (i.e., implicit spatial perspective chosen), and thereby requires disembodied, recomputing or cognitive control strategies^30^ to inhibit the interfering egocentric perspective during most trials. This mechanism is postulated to be reliant on areas of the CCN.

Subsequently, in further observing plausible effortful processing within the disembodied account at the behavioral level, RTs were elevated for the inconsistent subgroups than for the consistent subgroups, and results hinted at longer RTs for the subgroups choosing an altercentric perspective than those maintaining their own egocentric perspective. Furthermore, RT results at the individual trial level seem to be in line with the above neural trend (Fig. 8). Participants who irregularly undertook a spatial transformation for most of the trials (altercentric-inconsistent) were much slower to respond when switching to a non-transformation strategy (egocentric-response) than when deciding for a transformation strategy (altercentric-response). This may reflect a task-switching effect within this subgroup, whereby the cognitive process for selecting the ‘more difficult’ altercentric response involves suppressing that for an ‘easier’ egocentric response, leading to higher switch costs and longer reaction times for the latter. Conversely, such an effect is visibly weaker in the egocentric-inconsistent subgroup, since selecting the easier predominant response should not necessitate suppression of the cognitive process for a more difficult altercentric response.

Despite not being in the original hypotheses, it was only through the post-hoc introduction of the ‘consistency’ factor that the hypothesized differences between the accounts became more visible in the inconsistent subgroups of participants instead of at the whole-group level. Nevertheless, these neural and behavioral observations are ultimately based on 22 inconsistent participants (13 altercentric; 9 egocentric) out of a total of 117 participants. The above interpretation is not intended to be extended to the majority (95) of participants with consistent responses (31 altercentric; 64 egocentric). It should also be reiterated that in light of the exploratory nature of the study, reported statistical calculations pertaining to cortical activation are not confirmatory and are to be interpreted with caution. Multiple comparisons were uncorrected and reported results are for descriptive purposes only with no claims of generalizability. Furthermore, given the limited spatial resolution that fNIRS offers in measuring regions beyond the cortical surface, it is possible that there might be activation in subcortical regions mitigating this finding that we are unable to rule out.

Our exploratory neural and behavioral results partially support previous studies establishing a dissociable disembodied process that SPT can occur through without the need for an embodied mental transformation of the egocentric reference frame^2,4^. For instance, by collecting self-reported strategies to analyze performance on both an own-body transformation (OBT) perspective-taking task and response-inhibition task, Gardner and colleagues^2^ found that participants who took longer to change perspectives not only displayed delays in inhibiting spatially compatible responses but also reported that they adopted a left–right reversal strategy. This relationship between perspective-taking and response inhibition was absent in participants who reported employing the strategy of mentally transforming their own spatial orientation to that of the agent in the stimuli. At the neural level, our fNIRS findings allude to accounts that the lateral prefrontal cortex is key to the updating and regulation of responses, or for monitoring the initiation of inhibiting a competing response (perspective)^93–95^. Based on the descriptive patterns of activation in the left IFG and left DLPFC, our results hint that the inconsistent altercentric participants in our study may have relied on effortful, cognitive strategies such as re-computation of spatial relationships to select the altercentric perspective or active inhibition of the egocentric perspective^17^. The left lateralized activation in the IFG and DLPFC could also potentially speak for reliance on verbalization strategies (e.g., “Since the object is on my *left*, it must be on his/her *right*”) and spatial working memory in the decision-making process to support the transposing of ‘left’ and ‘right’ between the egocentric and altercentric perspectives^96^. However, as no self-reported strategy was collected from our participants, we cannot conclusively report which of the two strategies in the disembodied route was employed. Further neurophysiological research collecting strategy use data would be useful for elucidating the interaction between the DLPFC and IFG in implicit spatial perspective-taking.

Contrary to expectations that the mere presence of an agent performing a reaching action in the scene would prompt an equal if not greater proportion of altercentric than egocentric responses^1^, having fewer participants in the altercentric group meant a less prominent differentiation of strategies at the neural level. Unlike Mazarella et al.^24^ whose fMRI data evidenced greater activation in the left anterior inferior parietal sulcus (IPS), the posterior parts of the IFG, and the middle frontal gyrus (next to the premotor cortex) during altercentric perspective-taking processes, our lack of significant activation in the inferior parietal ROIs did not show support for an embodied processing route, which has been argued to underpin the link between action perception and visuospatial perspective-taking^3,4^. It is possible that participants either did not adopt the mental rotation-of-the-self strategy or that the static photographed stimuli were not sufficiently salient to substantially trigger embodied processing via the hMNS. To ensure that a response from the hMNS^97,98^ can be adequately evoked, further research could consider incorporating a traditional imitation task in the paradigm so that a comparison in brain activation between the SPT and imitation task can be compared and contrasted. In addition, it must be noted that Mazzarella et al.’s^24^ study involved explicit instructions to take a specific perspective, whereas we were interested in the neural substrates governing implicitly-chosen (i.e. self-selected) spatial perspectives. Giving participants the freedom to adopt any perspective they saw fit allowed us to evaluate a non-manipulated ‘natural perspective’. On the other hand, letting participants autonomously decide for one perspective or the other might be prone to extraneous influences such as a perceived expectation from the experimenter to answer in a specific way, mental state reasoning and social processing skills^22^. These may have implications on the underlying neural mechanisms (see Bukowski^99^ for a meta-analysis). To control for such factors, future research could consider including an additional task condition where explicit instructions to take different perspectives are imposed on participants, such that these perspectives would correspond either to the same or different perspective than the one they had adopted freely in the previous condition (baseline). This way, the neural underpinnings of any cost associated with adopting a more ‘unnatural perspective’ may be evaluated (refer to Arnold et al.^100^ for a similar design).

Our lack of evidence for the embodied route is also in contrast to studies such as Cavallo et al.^30^ which demonstrated, in a series of SPT experiments involving left/right, near/far and avatar/empty chair parameters, that participants remap spatial relations in a scene in accordance with the position of another’s in order to take an altercentric perspective. For instance, they found quicker egocentric judgments for objects presented nearer and to the right of participants, but comparably fast altercentric judgments for objects presented nearer and to the right of an avatar. The same RT pattern was observed when the avatar was replaced with an empty chair, but noticeably absent when neither an avatar nor an empty chair was present in the scene. By removing these affordances for perceived actions and, thus, for the potential embodied processing of spatial relations^9^, they critically demonstrated that the remapping strategy can be induced rapidly but selectively when an altercentric perspective is available to take. Their findings echo that of Tversky and Hard^1^, which showed that participants are willing to act on the more cognitively costly other-perspective as a response to subtle cues signaling a social interaction. According to Gunalp et al.^23^, different stimuli convey to the viewer different cues (e.g., social, directional, interactive) which can evoke individual differences during perspective-taking, especially in spatial orientation tests^62^. Human figures serve as social cues with agency and directionality (either front-facing or back-facing) as compared to symbolic objects such as arrows or chairs which have directionality but no agency. These cues are especially pertinent to strategy use in SPT, and potential engagement with an agentive social cue is more likely to elicit embodied strategies, such as bodily transformation, to complete tasks. Investigating SPT using inanimate objects with artificial agency in combination with fNIRS (e.g., front-facing human agent pointing versus front-facing physical object ‘pointing’) would be an interesting control condition for further research, to potentially single out the neural correlates of agency in the context of embodiment.

To that effect, the simple experimental design we adapted from Tversky and Hard^1^ engaged participants in a forced-choice scenario of having to implicitly decide between an egocentric and altercentric perspective whilst online processing was being measured by fNIRS. Both the presence of another person depicted in the stimuli and the lack of explicit demand to adopt a specific perspective offered the possibility to observe if participants would be encouraged to make a free and spontaneous decision to adopt the other-perspective. One of our hypotheses was that if the underlying mechanism driving altercentric SPT is governed by embodied processing, given the potential for action in the stimuli, there would be greater cortical activation in the areas associated with the hMNS for altercentric responses. The transformation strategies undertaken would then be embodied in nature, such as a mental rotation-of-the self. However, we acknowledge that to validate the underlying workings of such an embodied mechanism, we would have had to control for the influence of other variables such as the degree and axis of an imagined egocentric rotation or of the agent’s orientation (e.g., 40°/80°/120°/160°) in the experimental design, as in the studies by Mazarella et al.^24^ or Kozhevikov et al.^63^. Again, if participants had consciously used the mental rotation strategy, self-reported data on strategy use would have been useful for elucidating the brain-behavior relationships in the spatial judgment (decision-making) process. These are potential avenues for further research. Together with other methods such as eye-tracking in combination with cued retrospective verbal reports^101,102^, that is the playing back of own eye-movements to elicit retrospective verbalization of strategies during the task, differentiation between the embodied (remapping^30^) and disembodied (recomputing^30^) strategies at the neural level could be better measured and validated.

Whilst not a main focus of this paper, significantly lower PFT scores for the inconsistent subgroups than the consistent subgroups suggest that higher spatial abilities, as represented by spatial visualization, may promote one’s ability to maintain a chosen perspective during such an implicit SPT task. It is possible that this process is aided by an ability to efficiently acquire information from given pictorial visualizations^103^. Having a deeper understanding of the link between participants’ spatial abilities and their selected perspective at the neurophysiological level would have helped better elucidate the cognitive mechanisms behind SPT. Unfortunately, an observed lack of correlation between PFT scores and cortical activation in the ROIs is currently uninformative. To that end, we acknowledge that measuring spatial ability on the PFT alone may not be a sufficient measure for general spatial abilities which encompasses other factors apart from spatial visualization^104,105^. Furthermore, recent research has shown that the PFT does not measure spatial visualization exclusively but also reveals variation in strategies to paper-folding problems^106^. Hence, future work digging deeper into the role of spatial ability on perspective-taking should use a revised version of the PFT and also take into account a variety of tests to measure other aspects of spatial abilities. On a related note, sex differences have been documented to influence SPT ability but these differences may be dependent on social factors such as empathy rather than spatial abilities^107^. For example, although men are better at spatial-orientation and navigation tests, women are often seen as better empathizers than men^108^ which aid their ability to take the perspective of others in a social context (i.e., when they are told to take the perspective of a human figure)^107^. In light of this, despite having a majority of females among our participants, we did not detect any sex differences in performance among the four subgroups.

To the best of our knowledge, our study is the first to attempt employing fNIRS to predict the neural bases governing implicit altercentric SPT on the basis of cortical activation. By instructing participants to respond to queries strictly about the spatial relations between depicted objects, without any explicit instructions to adopt either an egocentric or altercentric reference frame, our exploratory post-hoc analyses allowed us to uncover inconsistency as a possibly modulating aspect of implicit altercentric spatial perspective-taking. The inconsistency dimension, and its interplay with perspective, sheds light on irregularity in the commitment to maintain a selected perspective as a potentially influencing factor in the effortful spatial transformation process. This is alluded to by greater neural activation in selective critical network regions belonging to the CCN and subsequently, a slower speed of decision-making (especially at the trial level). The current results of our hypotheses testing, whilst largely descriptive and partially conclusive, opens up the possibility for more confirmatory future research taking into account the following limitations. *Paradigm trials*—as prior studies show that perspective choices in the context of social interaction sometimes take longer to be worked out^109^, a greater number of trials (e.g. at least two repetitions of each trial) in the paradigm would provide a buffer for any mistakes and better allow for the distinguishing between deliberate and accidental switches. The 24 trials might have been too few to reliably categorize the participants into more homogenous groups and thus might explain why some of the differences in brain activation in the ROIs were only marginally significant. *Sample size*—the implicit nature of the paradigm encompasses a self-selecting element which increases the risk of group size variation and affects statistical power. Similar to the point above, increasing the sample size would increase the odds of a balanced group design for more reliable comparisons. *Instructions*—as aforementioned, including a condition with explicit instructions to take the egocentric or altercentric perspective would minimize the influence of unwanted extraneous factors and allow for a truer baseline measurement of spontaneous perspective-taking. *Strategy use*—as aforementioned, collecting self-reported post-experiment strategy use would be helpful for supplementing measured neurophysiological and behavior data, and elucidating the brain-behavior relationship. *fNIRS spatial resolution*—fNIRS is limited in its spatial resolution and restricts measurement to areas on the cortical surface (~ 1–2 cm). As such, activity in potentially crucial subcortical areas like the limbic system, orbitofrontal cortex and cingulate cortex cannot be measured and smaller areas of activation might be overlooked^110^. Although the ROIs in our study pertaining to the CCN and hMNS are mostly located in cortical regions, incorporating other neuroimaging techniques with better spatial resolution would provide a more accurate picture of activation patterns across the ROIs.

### Supplementary Information

Supplementary Information.

## Data Availability

The datasets generated during and/or analyzed during the current study are available from the corresponding author on reasonable request.

## References

[1] Tversky B, Hard BM (2009). Embodied and disembodied cognition: Spatial perspective-taking. Cognition.

[2] Gardner M, Brazier M, Edmonds C, Gronholm P (2013). Strategy modulates spatial perspective-taking: Evidence for dissociable disembodied and embodied routes. Front. Hum. Neurosci..

[3] Kessler K, Thomson LA (2010). The embodied nature of spatial perspective taking: Embodied transformation versus sensorimotor interference. Cognition.

[4] Kessler K, Rutherford H (2010). The two forms of visuo-spatial perspective taking are differently embodied and subserve different spatial prepositions. Front. Psychol..

[5] Surtees A, Apperly I, Samson D (2013). Similarities and differences in visual and spatial perspective-taking processes. Cognition.

[6] Surtees A, Apperly I, Samson D (2013). The use of embodied self-rotation for visual and spatial perspective-taking. Front. Hum. Neurosci..

[7] Levinson SC, Bloom P, Peterson M (1996). Language and Space.

[8] Levinson SC (2003). Space in Language and Cognition: Explorations in Cognitive Diversity.

[9] Creem-Regehr S, Gagnon K, Geuss M, Stefanucci J (2013). Relating spatial perspective taking to the perception of other's affordances: providing a foundation for predicting the future behavior of others. Front. Hum. Neurosci..

[10] Freundlieb M, Kovács ÁM, Sebanz N (2016). When do humans spontaneously adopt another’s visuospatial perspective?. J. Experim. Psychol. Hum. Percept. Perform..

[11] Mazzarella E, Hamilton A, Trojano L, Mastromauro B, Conson M (2012). Observation of another's action but not eye gaze triggers allocentric visual perspective. Q. J. Experim. Psychol..

[12] Surtees A, Apperly I, Samson D (2016). I’ve got your number: Spontaneous perspective-taking in an interactive task. Cognition.

[13] Vogeley K, Fink GR (2003). Neural correlates of the first-person-perspective. Trends Cogn. Sci..

[14] Mattan B, Quinn KA, Apperly IA, Sui J, Rotshtein P (2015). Is it always me first? Effects of self-tagging on third-person perspective-taking. J. Experim. Psychol. Learn. Memory Cogn..

[15] Keysar B, Barr DJ, Balin JA, Brauner JS (2000). Taking perspective in conversation: The role of mutual knowledge in comprehension. Psychol. Sci..

[16] Keysar B, Lin S, Barr DJ (2003). Limits on theory of mind use in adults. Cognition.

[17] Epley N, Keysar B, Van Boven L, Gilovich T (2004). Perspective taking as egocentric anchoring and adjustment. J. Personal. Soc. Psychol..

[18] Dumontheil I, Apperly IA, Blakemore S-J (2010). Online usage of theory of mind continues to develop in late adolescence. Dev. Sci..

[19] Ramsey R, Hansen P, Apperly I, Samson D (2012). Seeing it my way or your way: Frontoparietal brain areas sustain viewpoint-independent perspective selection processes. J. Cogn. Neurosci..

[20] Qureshi AW, Apperly IA, Samson D (2010). Executive function is necessary for perspective selection, not Level-1 visual perspective calculation: Evidence from a dual-task study of adults. Cognition.

[21] Samson D, Apperly IA, Braithwaite JJ, Andrews BJ, Bodley Scott SE (2010). Seeing it their way: Evidence for rapid and involuntary computation of what other people see. J. Experim. Psychol. Hum. Percept. Perform..

[22] Nielsen MK, Slade L, Levy JP, Holmes A (2015). Inclined to see it your way: Do altercentric intrusion effects in visual perspective taking reflect an intrinsically social process?. Q. J. Experim. Psychol..

[23] Gunalp P, Moossaian T, Hegarty M (2019). Spatial perspective taking: Effects of social, directional, and interactive cues. Memory Cogn.

[24] Mazzarella E, Ramsey R, Conson M, Hamilton A (2013). Brain systems for visual perspective taking and action perception. Social Neurosci..

[25] David N (2006). Neural representations of self versus other: Visual-spatial perspective taking and agency in a virtual ball-tossing game. J. Cogn. Neurosci..

[26] Wraga M, Shephard JM, Church JA, Inati S, Kosslyn SM (2005). Imagined rotations of self versus objects: An fMRI study. Neuropsychologia.

[27] Wraga M, Flynn CM, Boyle HK, Evans GC (2010). Effects of a body-oriented response measure on the neural substrate of imagined perspective rotations. J. Cogn. Neurosci..

[28] Vogeley K (2004). Neural correlates of first-person perspective as one constituent of human self-consciousness. J. Cogn. Neurosci..

[29] Kessler K, Wang H (2012). Spatial perspective taking is an embodied process, but not for everyone in the same way: Differences predicted by sex and social skills score. Spatial Cogn. Comput..

[30] Cavallo A, Ansuini C, Capozzi F, Tversky B, Becchio C (2017). When far becomes near: Perspective taking induces social remapping of spatial relations. Psychol. Sci..

[31] Lenggenhager B, Lopez C, Blanke O (2008). Influence of galvanic vestibular stimulation on egocentric and object-based mental transformations. Experim. Brain Res..

[32] Lambrey S, Doeller C, Berthoz A, Burgess N (2011). Imagining being somewhere else: Neural basis of changing perspective in space. Cerebral Cortex.

[33] Blanke O (2005). Linking out-of-body experience and self processing to mental own-body imagery at the temporoparietal junction. J. Neurosci..

[34] Zacks JM, Michelon P (2005). Transformations of visuospatial images. Behav. Cogn. Neurosci. Rev..

[35] Cross ES, Kraemer DJM, Hamilton AFDC, Kelley WM, Grafton ST (2009). Sensitivity of the action observation network to physical and observational Learning. Cerebral Cortex.

[36] van Gog T, Paas F, Marcus N, Ayres P, Sweller J (2009). The mirror neuron system and observational learning: Implications for the effectiveness of dynamic visualization. Educat. Psychol. Rev..

[37] Caramazza A, Anzellotti S, Strnad L, Lingnau A (2014). Embodied cognition and mirror neurons: A critical assessment. Ann. Rev. Neurosci..

[38] Fogassi L (2011). The mirror neuron system: How cognitive functions emerge from motor organization. J. Econ. Behav. Organ..

[39] Rizzolatti G, Sinigaglia C (2010). The functional role of the parieto-frontal mirror circuit: Interpretations and misinterpretations. Nat. Rev. Neurosci..

[40] Möttönen R, Farmer H, Watkins KE (2016). Neural basis of understanding communicative actions: Changes associated with knowing the actor’s intention and the meanings of the actions. Neuropsychologia.

[41] Gallese V, Sinigaglia C (2011). What is so special about embodied simulation?. Trends Cogn. Sci..

[42] Iacoboni M (2005). Grasping the intentions of others with one's own mirror neuron system. PLOS Biol..

[43] Rizzolatti G, Craighero L (2004). The mirror-neuron system. Annu. Rev. Neurosci..

[44] Geyer S, Matelli M, Luppino G, Zilles K (2000). Functional neuroanatomy of the primate isocortical motor system. Anat. Embryo..

[45] Keuken MC (2011). The role of the left inferior frontal gyrus in social perception: An rTMS study. Brain Res..

[46] Brucker B, Ehlis A-C, Häußinger FB, Fallgatter AJ, Gerjets P (2015). Watching corresponding gestures facilitates learning with animations by activating human mirror-neurons: An fNIRS study. Learn. Instr..

[47] Gronholm PC, Flynn M, Edmonds CJ, Gardner MR (2012). Empathic and non-empathic routes to visuospatial perspective-taking. Conscious. Cogn..

[48] May M, Wendt M (2012). Separating mental transformations and spatial compatibility effects in the own body transformation task. Cogn. Proc..

[49] Aichhorn M, Perner J, Kronbichler M, Staffen W, Ladurner G (2006). Do visual perspective tasks need theory of mind?. NeuroImage.

[50] Schurz M, Aichhorn M, Martin A, Perner J (2013). Common brain areas engaged in false belief reasoning and visual perspective taking: A meta-analysis of functional brain imaging studies. Front. Hum. Neurosci..

[51] Cole MW, Schneider W (2007). The cognitive control network: Integrated cortical regions with dissociable functions. NeuroImage.

[52] Nowinski WL (2021). Evolution of human brain atlases in terms of content, applications, functionality, and availability. Neuroinformatics.

[53] Schaefer A (2018). Local-global parcellation of the human cerebral cortex from intrinsic functional connectivity MRI. Cerebral Cortex.

[54] Yeo BTT (2011). The organization of the human cerebral cortex estimated by intrinsic functional connectivity. J. Neurophysiol..

[55] Ji JL (2019). Mapping the human brain's cortical-subcortical functional network organization. NeuroImage.

[56] Menon V, D’Esposito M (2022). The role of PFC networks in cognitive control and executive function. Neuropsychopharmacology.

[57] Szczepanski SM, Pinsk MA, Douglas MM, Kastner S, Saalmann YB (2013). Functional and structural architecture of the human dorsal frontoparietal attention network. Proc. Natl. Acad. Sci..

[58] Miller EK, Cohen JD (2001). An integrative theory of prefrontal cortex function. Ann. Rev. Neurosci..

[59] Aron AR, Robbins TW, Poldrack RA (2004). Inhibition and the right inferior frontal cortex. Trends Cogn. Sci..

[60] Aron AR, Robbins TW, Poldrack RA (2014). Inhibition and the right inferior frontal cortex: One decade on. Trends Cogn. Sci..

[61] Depue BE, Orr JM, Smolker HR, Naaz F, Banich MT (2016). The organization of right prefrontal networks reveals common mechanisms of inhibitory regulation across cognitive, emotional, and motor processes. Cerebral Cortex.

[62] Hegarty M, Waller D (2004). A dissociation between mental rotation and perspective-taking spatial abilities. Intelligence.

[63] Kozhevnikov M, Motes MA, Rasch B, Blajenkova O (2006). Perspective-taking vs. mental rotation transformations and how they predict spatial navigation performance. Appl. Cogn. Psychol..

[64] Ekstrom RB, French JW, Harman HH, Dermen D (1976). Manual for Kit of Factor-Referenced Cognitive Tests.

[65] Brucker, B., de Koning, B. B., Ehlis, A.-C., Rosenbaum, D. & Gerjets, P. in *39th Annual Conference of the Cognitive Science Society (Cogsci 2017)* (London, UK, 2017).

[66] Haeussinger FB (2014). Reconstructing functional near-infrared spectroscopy (fNIRS) signals impaired by extra-cranial confounds: An easy-to-use filter method. NeuroImage.

[67] Haeussinger FB (2011). Simulation of near-infrared light absorption considering individual head and prefrontal cortex anatomy: Implications for optical neuroimaging. PLoS One.

[68] Soltanlou M, Sitnikova MA, Nuerk H-C, Dresler T (2018). Applications of functional near-infrared spectroscopy (fNIRS) in studying cognitive development: The case of mathematics and language. Front. Psychol..

[69] Rosenbaum D (2020). Neuronal correlates of spider phobia in a combined fNIRS-EEG study. Sci. Rep..

[70] Jasper HH (1958). The ten-twenty electrode system of the International Federation. Electroencephalogr. Clin. Neurophysiol..

[71] Tsuzuki D (2007). Virtual spatial registration of stand-alone fNIRS data to MNI space. NeuroImage.

[72] Rorden C, Brett M (2000). Stereotaxic display of brain lesions. Behav. Neurol..

[73] Singh AK, Okamoto M, Dan H, Jurcak V, Dan I (2005). Spatial registration of multichannel multi-subject fNIRS data to MNI space without MRI. NeuroImage.

[74] Tsuzuki D, Dan I (2014). Spatial registration for functional near-infrared spectroscopy: From channel position on the scalp to cortical location in individual and group analyses. NeuroImage.

[75] Tzourio-Mazoyer N (2002). Automated anatomical labeling of activations in SPM using a macroscopic anatomical parcellation of the MNI MRI single-subject brain. NeuroImage.

[76] Rosenbaum D (2018). Comparison of speed versus complexity effects on the hemodynamic response of the trail making test in block designs. Neurophotonics.

[77] Rosenbaum D (2018). Cortical hemodynamic changes during the trier social stress test: An fNIRS study. NeuroImage.

[78] Cui X, Bray S, Reiss AL (2010). Functional near infrared spectroscopy (NIRS) signal improvement based on negative correlation between oxygenated and deoxygenated hemoglobin dynamics. NeuroImage.

[79] Scholkmann F (2014). A review on continuous wave functional near-infrared spectroscopy and imaging instrumentation and methodology. NeuroImage.

[80] Tachtsidis I, Scholkmann F (2016). False positives and false negatives in functional near-infrared spectroscopy: Issues, challenges, and the way forward. Neurophotonics.

[81] Brigadoi S (2014). Motion artifacts in functional near-infrared spectroscopy: A comparison of motion correction techniques applied to real cognitive data. NeuroImage.

[82] Zhang X, Noah JA, Hirsch J (2016). Separation of the global and local components in functional near-infrared spectroscopy signals using principal component spatial filtering. Neurophotonics.

[83] Plichta MM, Heinzel S, Ehlis AC, Pauli P, Fallgatter AJ (2007). Model-based analysis of rapid event-related functional near-infrared spectroscopy (NIRS) data: A parametric validation study. NeuroImage.

[84] Hahn T (2011). Neurovascular coupling in the human visual cortex is modulated by cyclooxygenase-1 (COX-1) gene variant. Cerebral Cortex.

[85] Boden S (2007). The oxygenation response to functional stimulation: Is there a physiological meaning to the lag between parameters?. NeuroImage.

[86] Leff DR (2011). Assessment of the cerebral cortex during motor task behaviours in adults: A systematic review of functional near infrared spectroscopy (fNIRS) studies. NeuroImage.

[87] R: A language and environment for statistical computing (R Foundation for Statistical Computing, Vienna, Austria, 2018).

[88] Berry D (2017). A p-value to die for. J. Am. Stat. Assoc..

[89] Hershberger, S. L. in *Encyclopedia of Statistics in Behavioral Science* (eds B. S. Everitt & D. C. Howell) (2005).

[90] Bates D, Maechler M, Bolker B, Walker S (2015). Fitting linear mixed-effects models using lme4. J. Stat. Softw..

[91] Nakagawa S, Schielzeth H (2013). A general and simple method for obtaining *R*^*2*^ from generalized linear mixed-effects models. Methods Ecol. Evol..

[92] Nakagawa S, Johnson PCD, Schielzeth H (2017). The coefficient of determination *R*^*2*^ and intra-class correlation coefficient from generalized linear mixed-effects models revisited and expanded. J. R. Soc. Interface.

[93] Samson D, Apperly IA, Kathirgamanathan U, Humphreys GW (2005). Seeing it my way: A case of a selective deficit in inhibiting self-perspective. Brain.

[94] Vogeley K (2001). Mind reading: Neural mechanisms of theory of mind and self-perspective. NeuroImage.

[95] van der Meer L, Groenewold NA, Nolen WA, Pijnenborg M, Aleman A (2011). Inhibit yourself and understand the other: Neural basis of distinct processes underlying Theory of Mind. NeuroImage.

[96] Reuter-Lorenz PA (2000). Age differences in the frontal lateralization of verbal and spatial working memory revealed by PET. J. Cogn. Neurosci..

[97] Iacoboni M (2005). Neural mechanisms of imitation. Curr. Opin. Neurobiol..

[98] Cattaneo L, Rizzolatti G (2009). The mirror neuron system. Arch. Neurol..

[99] Bukowski H (2018). The neural correlates of visual perspective taking: A critical review. Curr. Behav. Neurosci. Rep..

[100] Arnold G, Spence C, Auvray M (2016). Taking someone else’s spatial perspective: Natural stance or effortful decentring?. Cognition.

[101] van Gog T, Paas F, van Merriënboer JJG, Witte P (2005). Uncovering the problem-solving process: Cued retrospective reporting versus concurrent and retrospective reporting. J. Experim. Psychol. Appl..

[102] Eger, N., Ball, L. J., Stevens, R. & Dodd, J. in *Proceedings of HCI 2007 The 21st British HCI Group Annual Conference University of Lancaster, UK 21.* 1–9.

[103] Höffler TN (2010). Spatial ability: Its influence on learning with visualizations—A meta-analytic review. Educat. Psychol. Rev..

[104] Carroll JB (1993). Human Cognitive Abilities: A Survey of Factor-Analytic Studies.

[105] Lee-Cultura S, Giannakos M (2020). Embodied interaction and spatial skills: A systematic review of empirical studies. Interact. Comput..

[106] Burte H, Gardony AL, Hutton A, Taylor HA (2019). Knowing when to fold 'em: Problem attributes and strategy differences in the Paper Folding test. Personal. Indiv. Differ..

[107] Tarampi MR, Heydari N, Hegarty M (2016). A tale of two types of perspective taking: Sex differences in spatial ability. Psychol. Sci..

[108] Baron-Cohen S (2002). The extreme male brain theory of autism. Trends Cogn. Sci..

[109] Roche, J., Dale, R. & Kreuz, R. J. in *Proceedings of the Annual Meeting of the Cognitive Science Society.* 206–211.

[110] Burns, S. M. & Lieberman, M. D. The use of functional near infrared spectroscopy (fNIRS) for unique contributions to social and affective neuroscience. psyarxiv.com/kygbm [Preprint]. (2019 [cited 2019 July 16]). https://psyarxiv.com/kygbm.

