# Supplementary material for: Exploring the neural basis and modulating factors of implicit altercentric spatial perspective-taking with fNIRS
Source: Sci Rep. 2023 Nov 23;13:20627. doi: 10.1038/s41598-023-46205-w (PMC10667356; doi:10.1038/s41598-023-46205-w)
Supplement: Supplementary file 1 — Supplementary Information. [file 41598_2023_46205_MOESM1_ESM.pdf]

# Supplementary Material

**Title**

Exploring the neural basis and modulating factors of implicit altercentric spatial perspective-taking with fNIRS

**Authors**

Natania Ang[1,*ꜜ], Birgit Brucker[2ꜜ], David Rosenbaum[3], Martin Lachmair[4], Thomas Dresler[1,3], Ann-Christine Ehlis[1,3] & Peter Gerjets[1,2]

**Affiliations**

[1]LEAD Graduate School and Research Network, University of Tübingen, Walter-Simon-Straße 12, Tübingen, 72072, Germany

[2]Leibniz-Institut für Wissensmedien, Schleichstraße 6, Tübingen, 72076, Germany

[3]Department of Psychiatry and Psychotherapy, Tübingen Center for Mental Health, University Hospital Tübingen, Calwerstraße 14, Tübingen, 72076, Germany

[4]Duale Hochschule Baden-Württemberg Villingen-Schwenningen, Karlstraße 29, Villingen-Schwenningen, 78054, Germanyy

[*]Natania Ang; natania-shuyi.ang@uni-tuebingen.de

[ꜜ]These authors contributed equally to this work

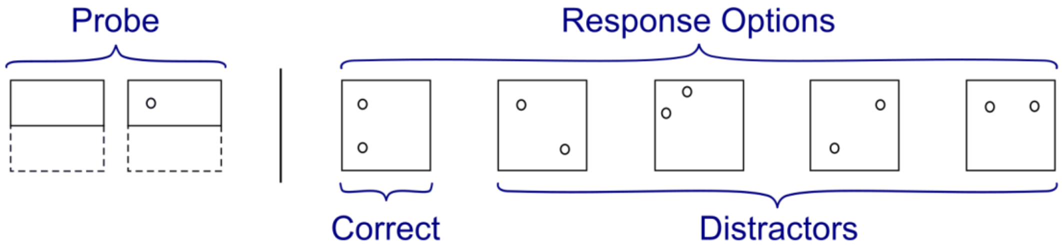


Supplementary Fig. S1. Example of test item from the short version of the Paper Folding Test (PFT). Each item consists of a probe and its five corresponding response options, of which one is correct and the remaining are distractors. [Figure adapted from the VZ-2 Paper Folding Test and created with Inkscape version 0.92.5. https://www.inkscape.org]

Supplementary Table S1. Summary of results from the linear mixed-effects model.

| **Fixed effects** | **Est.** | **SE** | ***t*** | ***p*** | **95% CI** |
|---|---|---|---|---|---|
| *Intercept* | 8.107 | 0.101 | 80.360 | < .001 | [7.903, 8.307] |
| Group | 0.161 | 0.157 | 1.021 | .316 | [-0.167, 0.480] |
| Response | 0.233 | 0.082 | 2.836 | .010 | [0.047, 0.405] |
| Group*Response | -0.497 | 0.127 | -3.905 | .001 | [-0.746, -0.241] |

| **Random effects** | **Variance** | **SD** | **Correlation** |
|---|---|---|---|
| Trial (Intercept) | 0.043 | 0.206 | |
| Subject (Intercept) | 0.102 | 0.320 | |
| Subject*Response | 0.033 | 0.182 | -0.10 |
| Residual | 0.135 | 0.368 | |

Number of observations: 521; 24 trials; 22 participants; Model formula: RTlog ~ Group*Response + (1 + Response|Subject) + (1|Trial)
